# RNA-Seq analysis reveals the growth and photosynthetic responses of rapeseed (*Brassica napus* L.) under red and blue LEDs with supplemental yellow, green, or white light

**DOI:** 10.1038/s41438-020-00429-3

**Published:** 2020-12-01

**Authors:** Xiaoying Liu, Zheng Chen, Mohammad Shah Jahan, Yixuan Wen, Xuyang Yao, Haifeng Ding, Shirong Guo, Zhigang Xu

**Affiliations:** 1 College of Agriculture, Nanjing Agricultural University, 210095 Nanjing, China; 2 College of Horticulture, Nanjing Agricultural University, 210095 Nanjing, China; 3 Department of Horticulture, Sher-e-Bangla Agricultural University, Dhaka, 1207 Bangladesh; 4 College of Agriculture, Shihezi University, 832003 Shihezi, China

**Keywords:** Light responses, Plant physiology

## Abstract

Compound light is required for plant growth and development, but the response mechanisms of plants are undercharacterized and not fully understood. The present study was undertaken to evaluate the effect of supplemental light (green light, G; white light, W; yellow light, Y) added to red–blue light (RB) and sole W on the growth and photosynthesis of rapeseed seedlings. The results revealed that supplemental G/W improved the growth and photosynthesis of seedlings, but supplemental Y significantly reduced the photosynthetic rate and palisade tissue layer. Sole W caused similar responses in terms of growth, leaf development, oxidative damage, and antioxidant capability as supplemental Y. In total, 449, 367, 813, and 751 differentially expressed genes (DEGs) were identified under supplemental G, Y, and W and sole W, respectively, compared to RB. The DEGs under different lights were closely associated with pathways such as light stimulus and high-light response, root growth, leaf development, photosynthesis, photosynthesis-antenna proteins, carbohydrate synthesis and degradation, secondary metabolism, plant hormones, and antioxidant capacity, which contributed to the distinct growth and photosynthesis under different treatments. Our results suggest that Y is more likely substituted by other wavelengths to achieve certain effects similar to those of supplemental Y, while G has a more distinctive effect on rapeseed. Taken together, supplementation RB with G/W promotes the growth of rapeseed seedlings in a controlled environment.

## Introduction

Light, as a vital environmental factor and energy source, governs plant growth throughout the lifecycle^1^. Via long-term evolution, plants have adapted to the broad spectrum of light in the natural environment. Under natural conditions, plants not only absorb visible light, including light in the red (620–750 nm) and blue (400–500 nm) regions, by utilizing photosynthetic pigments but also recognize invisible light via photoreceptors, which directly control photomorphogenesis and plant growth^2^. The effects of light from a large range of wavebands on plant life are largely driven by red/far-red and ultraviolet-A (UV-A)/blue wavebands of the electromagnetic spectrum, and thus, a mixture of red and blue spectral components is required for the normal growth and development of plants^2,3^. Compared to full-spectrum white light or red–blue light (RB), monochromatic wavebands usually have negative effects on the growth and physiological performance of plants, resulting in, for example, abnormal leaf morphology^4^, reduced photosynthetic rate and Rubisco activity^5^, reduced biomass and specific leaf area^6^, and reduced carbohydrate accumulation and quantum yield of photosystem II (PSII)^7^. The effect of monochromatic blue light in higher plants is controversial, as blue light can also achieve growth comparable to that observed under white light^8^ and can even induce significant promotion of plant growth compared to broad-spectrum light^9,10^. Regardless, the region of the spectrum containing blue light is essential for the normal growth of plants^11,12^.

In terms of monochromatic light, visible light (except for red and blue wavebands), green/yellow light (500–600 nm) in particular, has been traditionally considered to be developmentally inconsequential because of the very limited absorption of this light by plants, which has led to very little attention being paid to the effects of green/yellow light on plant growth and development for a long time^2,3^. With the emergence of light-emitting diodes (LEDs) and their wide application in horticultural facilities, the functions of green/yellow light have been gradually revealed using solid-state lighting systems. Folta and Maruhnich^3^ summarized the extensive regulatory roles of “green light” (500–600 nm) in plant growth and development, drawing from data gathered over the past 50 years of plant photobiological research. They concluded that “green light” sensory systems adjusted growth and development by interacting with red and blue sensors. Here, we should emphasize that, in that review^3^, the range of “green light” was extended to include the green (500–570 nm) and yellow (570–600 nm) sections of the spectrum. Folta and Maruhnich^3^ indicated that monochromatic green/yellow light hindered the growth and development of plants. Recent results further showed that the quantum yield of green/yellow light for photosynthesis was much lower than that of RB, and monochromatic or broadband green light acted as an inhibitor of the growth and development of plants^5,13^. It was reported that monochromatic green light caused considerable reversal of blue light-stimulated stomatal opening^14,15^, resulting in a reduced photosynthetic rate^5,7^ and a significant decrease in plant weight^16,17^. In addition, monochromatic green light primarily triggered responses associated with the shade avoidance syndrome, such as seedling spindling and reduced leaf investment^8,13^. The difference in peak wavelength among the green LEDs also induced distinct growth responses, and compared to long-wavelength green light, short-wavelength green light was available for active plant growth^17,18^. There have been far fewer reports about monochromatic yellow light than about green light. Several reports have shown that yellow light reduces the net photosynthetic rate (Pn) and chlorophyll fluorescence parameters of Welsh onion^5^ and the carbohydrate accumulation and Rubisco activity of cucumber^7^, and a high amount of yellow light does not favor microtuber formation and growth^19^ and worsens the growth of *Epimedium pseudowushanense*, but promotes the accumulation of bioactive flavonoids in this plant^6^.

In recent years, with a deeper understanding of the effect of the monochromatic spectrum on the life activities of plants, it has gradually been found that mixed irradiation has a great potential to facilitate plant growth and development. Although the quantum yield of green/yellow light for photosynthesis is quite low, plants can absorb 43% to 87% of the green light^20^ and efficiently use this part of the energy for photosynthesis at the inner canopy level and in deeper layers of the leaf mesophyll^3,21^, and thus, supplemental green light (only at low levels) based on compound light has been shown to promote growth in lettuce^22^, sweet pepper^23^, and cucumber^24^. In addition, supplementing blue and/or red light with green light can also improve nutritional quality in lettuce^22,25^. Kim et al.^22,26^ reported that the addition of waveband sections (500–600 nm) at lower input levels to red–blue LEDs had positive effects on lettuce morphology and dry matter accumulation. These results suggest that the green/yellow wavelengths should be considered important factors affecting plant development. In addition, different supplemental lights, including the full spectrum, have been reported to increase the nutritional quality of lettuce^27^. However, information regarding which supplemental light is a better strategy for plant growth in a controlled environment is insufficient. Therefore, it is important to make efforts to provide new insight into this issue.

For the determination of the optimal light conditions for plant growth, it is critical to understand the effect of monochromatic light at narrow bandwidths on plant growth and development at a molecular level^28^. More importantly, under light conditions meeting the basic light requirement for plant growth and exploring the effects of supplemental light is a better strategy for plant production, as well as understanding the cross-talk among lights. The cross-talk among different light parameters is complex, and the response mechanisms of plants are not yet understood; thus, the regulatory network still needs to be elucidated at the physiological and molecular levels. RNA-sequencing (RNA-Seq), a high-throughput technology, facilitates the systematic monitoring of cellular responses in the transcriptome and has been applied in some studies on light responses^6,28^. The effect of a combination of red and blue LEDs on photosynthesis and chlorophyll and carotenoid (Car) biosynthesis in *Brassica campestris* L. has also been explored using RNA-Seq technology^29^. However, the effects and molecular mechanism of supplemental light added to RB on plants, including rapeseed, remain undercharacterized and poorly understood.

LEDs can provide a better combination of the visible spectrum than other light sources, allowing researchers to obtain comprehensive and precise insight into the effects of different regions of the spectrum or specific wavelengths on plants^30^. In this study, using LEDs as a light source, we selected rapeseed (*B. napus* L.) to test its adaptive responses, in terms of growth traits, photosynthetic characteristics, oxidative damage, and antioxidant capability, to supplementary yellow, green, or white lights (Y, G, or W) added to RB, and we used RNA-Seq technology to reveal the response mechanisms by identifying differentially expressed genes (DEGs) and performing Gene Ontology (GO) and Kyoto Encyclopedia of Genes and Genomes (KEGG) enrichment analyses of the DEGs. Our data indicated that the DEGs were associated with pathways, including light stimulus and high-light response, root and leaf development, photosynthesis, carbohydrate synthesis and degradation, secondary metabolism, plant hormones, and antioxidant capacity, that contributed to the distinct growth and photosynthesis of rapeseed under different lights.

## Results

### Effects of supplemental light on growth traits and root activity

**Fig. 1:**
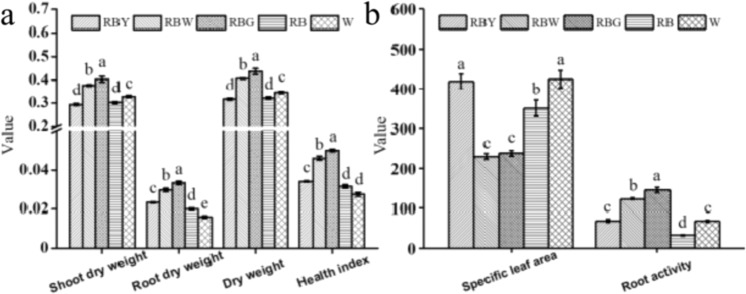
Growth traits and root activity of rapeseed seedlings under different lights. The units of weight, specific leaf area, and root activity are “g,” “cm^2^ g^−1^,” and “µg g^−1^ h^−1^,” respectively. RB red–blue light, RBG red–blue–green light, RBW red–blue–white light, RBY red–blue–yellow light, W white light. Vertical bars are means ± SDs (*n* = 3). Bars labeled with lowercase letters are significantly different by Duncan’s test at the *P* < 0.05 level

The growth traits and root activity of rapeseed seedlings grown under sole W, RB, and RB with supplemental Y, W, or G (i.e., RBY, RBW, and RBG) were significantly different (Fig. 1). Compared to RB, supplemental W/G significantly increased dry weight (DW), health index, and root activity, but decreased specific leaf area, while supplemental Y induced a negligible effect on dry matter accumulation in shoots, but enhanced root growth (higher root DW and root activity) and specific leaf area, suggesting that supplemental W/G facilitates whole-plant growth, while supplemental Y mainly contributes to local regions, such as roots and leaves. Sole W increased the root activity as well as shoot and plant DW and specific leaf area, but decreased the health index compared to RB (Fig. 1).

### Effects of supplemental light on leaf anatomical and stomatal traits

**Fig. 2:**
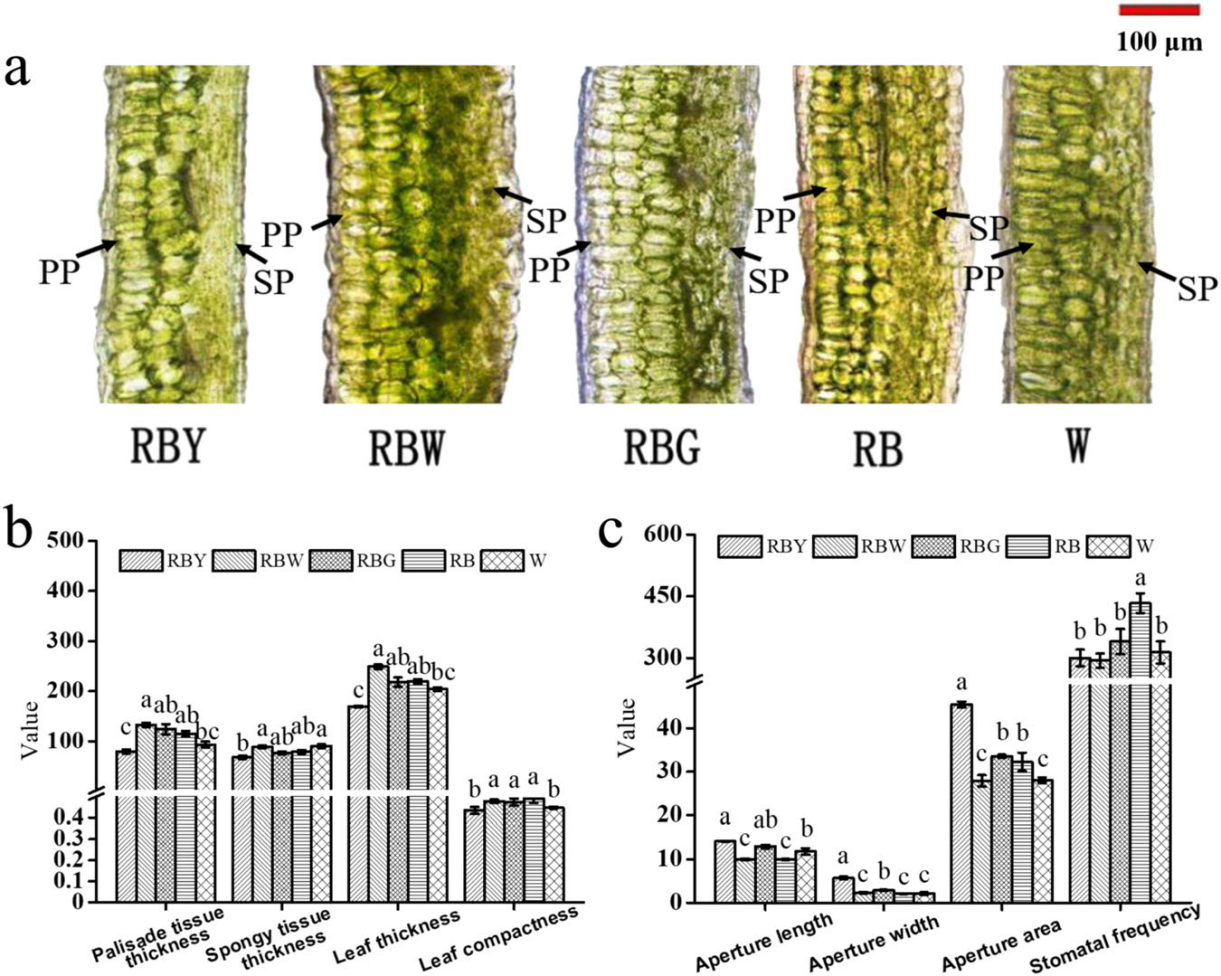
The effect of light quality on leaf development. Leaf anatomical phenotype (**a**) and traits (**b**) and lower epidermal stomata (**c**) of rapeseed seedlings under different lights. PP palisade parenchyma, SP spongy parenchyma, RB red–blue light, RBG red–blue–green light, RBW red–blue–white light, RBY red–blue–yellow light, W white light. The units of the thickness of palisade tissue, spongy tissue and leaf and aperture length and aperture width are “µm”; the units of aperture area and stomatal frequency are “µm^2^” and “No. mm^−2^,” respectively. Vertical bars are means ± SDs (four views of each replicate, *n* = 3). Bars labeled with lowercase letters are significantly different by Duncan’s test at the *P* < 0.05 level

The leaf is a major photosynthetic site, and leaf anatomy and stomata were observed to analyze the effects of supplemental light on leaf development (Fig. 2). The anatomical results showed that the leaves grown under RB and supplemental G/W induced three layers of palisade cells, whereas those grown under sole W and supplemental Y both had only two layers, leading to their lower anatomical parameters, including leaf thickness and leaf compactness (Fig. 2a, b). However, stomatal size significantly increased under supplemental Y/G relative to RB and other lights, while supplemental W and sole W resulted in a smaller aperture area. Stomatal frequencies were reduced under all supplemental lights compared to RB (Fig. 2c).

### Effects of supplemental light on Pn, chlorophyll fluorescence, and photosynthate

**Fig. 3:**
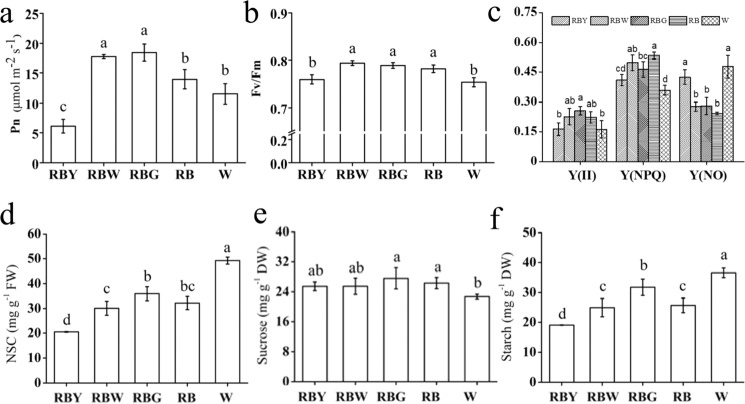
The effect of light quality on the photosynthetic characteristics. Net photosynthetic rate (Pn, **a**); chlorophyll fluorescence parameters, including Fv/Fm (**b**), Y(II), Y(NPQ), and Y(NO) (**c**) in the top third leaf of rapeseed; and the levels of NSC (**d**), sucrose (**e**), and starch (**f**) under different lights. RB red–blue light, RBG red–blue–green light, RBW red–blue–white light, RBY red–blue–yellow light, W white light. Vertical bars are means ± SDs (*n* = 3). Bars labeled with lowercase letters are significantly different by Duncan’s test at the *P* < 0.05 level

Leaves grown under supplemental W/G presented a higher Pn value than those grown under RB, and the opposite was observed for those grown under supplemental Y (Fig. 3a). Supplemental Y and sole W resulted in similar reductions in the maximum photochemical efficiency of PSII (Fv/Fm), non-photochemical quenching coefficient (qN), quantum yield of regulated energy dissipation [Y(NPQ)], and relative electron transfer rate of PSII (rETR) of rapeseed and a similar increase in the quantum yield of non-regulated energy dissipation [Y(NO)], while these photosynthetic characteristics under supplemental W/G were more analogous to those under RB treatment (Fig. 3b, c and Supplementary Fig. S1a, b). The results implied that the lower photosynthetic rate observed with supplemental Y and sole W might have contributed to the inhibition of PSII activity.

We determined the levels of sucrose, starch, and non-structural carbohydrates (NSCs) during the daytime (i.e., 0.5 light period) (Fig. 3d–f). Although the sucrose content under three supplemental lights was almost equivalent to that under RB, the levels of NSC and starch under supplemental Y were significantly lower than those under other treatments, while the levels of NSC and starch were the highest, but the sucrose content was the lowest, in seedlings exposed to sole W. To better understand the dynamic accumulation of photosynthate, leaf discs were stained with an iodine solution to evaluate starch storage before and after daytime. Supplementary Fig. S2a–c shows that there was no significant difference in the starch content among the three supplemental light treatments and the RB treatment after the daytime, but supplemental G induced higher starch accumulation than the other treatments before the daytime, suggesting that supplemental G degrades the lowest amount of starch during the night-time.

### Effects of supplemental light on oxidative damage and antioxidant capability

**Fig. 4:**
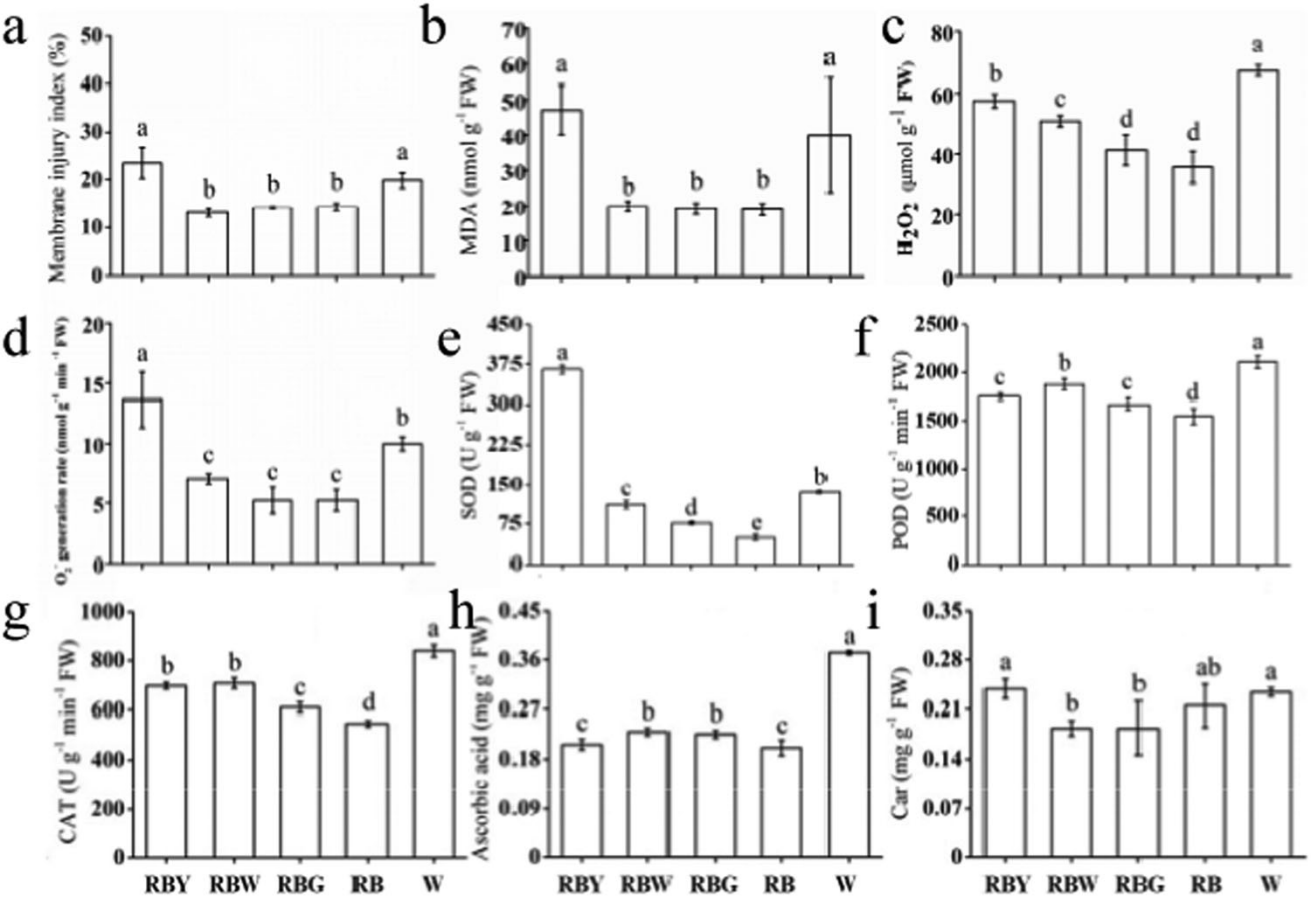
The effect of light quality on the oxidative damage and antioxidant capability. Membrane injury index (**a**), MDA content (**b**), ROS levels (**c**, **d**), and antioxidant capacity (**e**–**i**) in leaves of rapeseed under different lights. Car carotenoids, CAT catalase, MDA malondialdehyde, POD peroxidase, SOD superoxide dismutase, RB red–blue light, RBG red–blue–green light, RBW red–blue–white light, RBY red–blue–yellow light, W white light. Vertical bars are means±SDs (*n* = 3). Bars labeled with lowercase letters are significantly different by Duncan’s test at the *P* < 0.05 level

A reduction in Fv/Fm indicates that the plant is suffering from a suboptimal environment, so we determined the oxidative damage and antioxidant capability of rapeseed. Compared with RB, supplemental Y and sole W significantly increased the membrane injury index, malondialdehyde (MDA) content, and the levels of reactive oxygen species (ROS), including O_2_^−^ and H_2_O_2_, while supplemental W/G increased only the H_2_O_2_ level (Fig. 4a–d). For the antioxidant enzyme system, all treatments substantially increased the superoxide dismutase (SOD), peroxidase (POD), and catalase (CAT) activities compared to RB. The SOD activity was 6.24-fold higher under supplemental Y than under RB, and sole W induced the highest increases in POD and CAT activities (Fig. 4e–g). In addition, sole W and supplemental W/G significantly increased the ascorbic acid (AsA) content, while supplemental Y did not, compared to RB, and the supplemental Y and sole W treatment groups had a higher level of Car than the supplemental W/G treatment group (Fig. 4h, i). These results indicated that ROS homeostasis in rapeseed under supplemental Y and sole W is disturbed, and antioxidant components are conspicuously induced to scavenge excessive oxidation active substances.

### Identification of DEGs under different light treatments

To gain insight into the mechanisms underlying the supplemental light responses, messenger RNA (mRNA) sequencing of the leaf samples after 15 days of irradiation was conducted. Differences in gene expression were examined to analyze the genes that may participate in light-induced morphological alteration. To evaluate the reliability of the RNA-Seq data, 16 randomly selected DEGs were used to determine the gene expression level by quantitative real-time polymerase chain reaction (qRT-PCR). The gene expression patterns obtained with the two methods showed similar trends, validating the reliability of the RNA-Seq results (Supplementary Fig. S3).

**Fig. 5:**
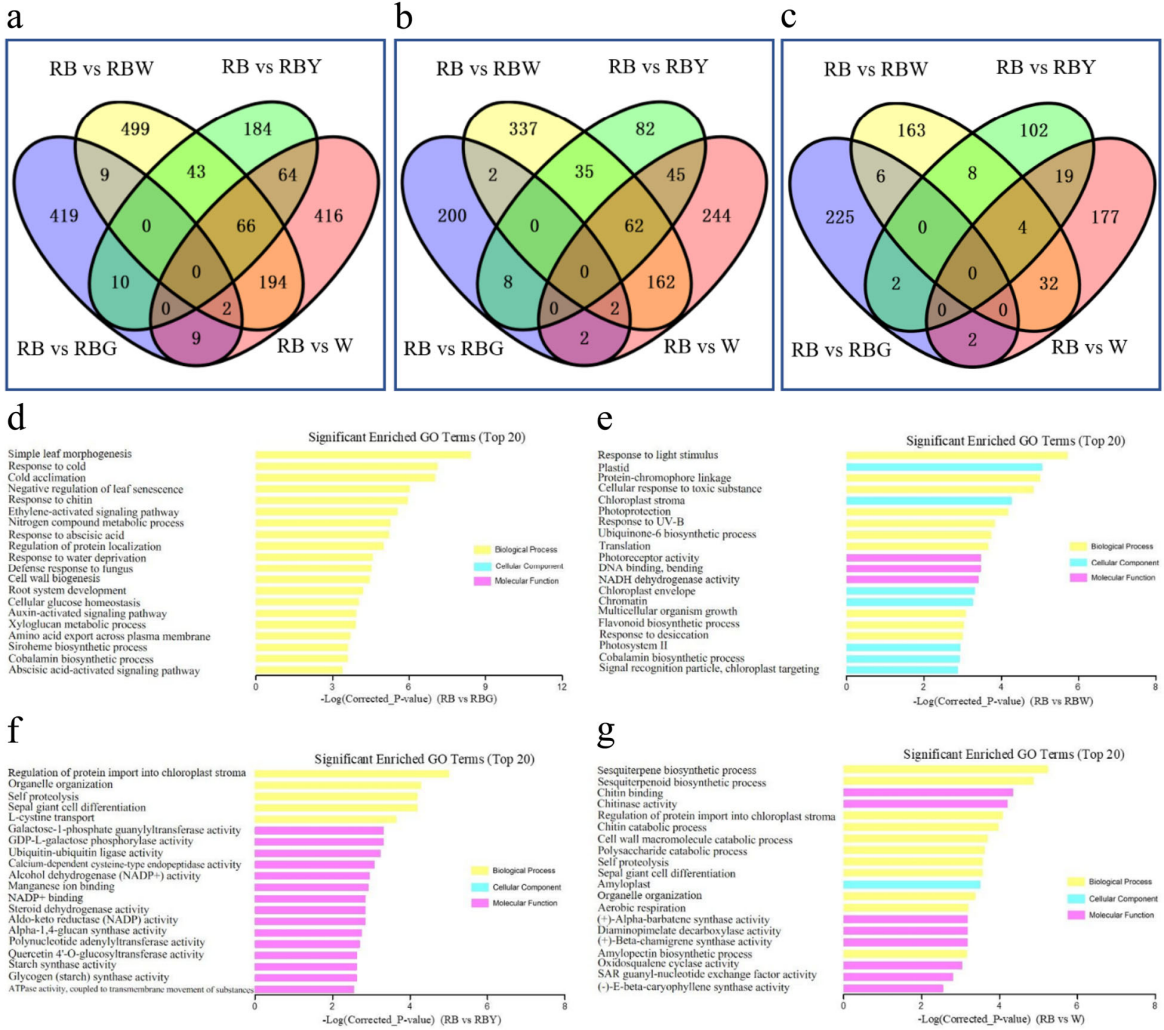
Venn diagram and top 20 significantly enriched GO terms of the differentially expressed genes (DEGs). **a** Total DEGs, **b** upregulated DEGs, **c** downregulated DEGs; **d**–**g** top 20 significantly enriched GO terms in the RB vs. RBG, RB vs. RBW, RB vs. RBY, and RB vs. W comparisons at the *P* < 0.05 level. DEGs screened with |log_2_(FC) | ≥ 1.0, *P* < 0.05 and false discovery rate (FDR) < 0.05 among four pairwise comparisons. FC fold change, RB red–blue light, RBG red–blue–green light, RBW red–blue–white light, RBY red–blue-yellow light, W white light

Through pairwise comparisons, a total of 449, 813, 367, and 751 DEGs were identified between RB and RBG, RB and RBW, RB and RBY, and RB and W, respectively (Fig. 5a). Among these DEGs, 214 genes were upregulated and 235 genes were downregulated under RBG compared with RB; 600 genes were upregulated and 213 genes were downregulated under RBW compared with RB; 232 genes were upregulated and 135 genes were downregulated under RBY compared with RB; 517 genes were upregulated and 234 genes were downregulated under sole W compared with RB (Fig. 5b, c). Comparison of expression patterns revealed that four upregulated DEGs and six downregulated DEGs overlapped between RB vs. RBG and RB vs. RBW (Fig. 5b, c and Supplementary Table S1).

### GO category analysis of light-responsive genes

We used GO assignments to classify the functions of the DEGs. The enriched genes in four comparisons were annotated in three main GO categories, including “biological process (BP)”, “cellular component (CC),” and “molecular function (MF).” The top 20 GO enrichment terms were almost completely different between the four comparisons (Fig. 5d–g and Supplementary Table S2). In the comparison of RB vs. RBG, the functions of all the DEGs were enriched in BP, and the significantly enriched GO terms were related to the regulation of leaf development, response to stress, cell wall formation, root system development, and plant hormone signal process (Fig. 5d). In the comparison of RB vs. RBW, the enriched GO terms are associated with chloroplast-related components, light-responsive processes and activities, respiratory chain activity, and multicellular organism growth (Fig. 5e). In the comparisons of RB vs. RBY and RB vs. W, four common BP terms, namely, regulation of protein import into chloroplast stroma, organelle organization, self-proteolysis, and sepal giant cell differentiation, were significantly enriched. The rest of the top GO terms in the RB vs. RBY comparison were mostly classified into the MF category and were related to carbohydrate metabolism, protein degradation, and biotic stress-responsive enzyme activities, while the majority of the top GO terms in the RB vs. W comparison were involved in sesquiterpenoid and triterpenoid biosynthesis, polysaccharide synthesis and catabolism, and cell wall macromolecule catabolism (Fig. 5g).

To gain a deeper understanding of the light-induced regulation of BPs related to leaf development, light stimuli, chlorophyll, carbohydrates, plant hormones, and oxidative stress, the enriched GO terms were analyzed, and terms for which the enrichment factor was >2 and *P* values were <0.01 are listed in Table S3. The GO terms related to response to abscisic acid and high light intensity, Car catabolism process, leaf senescence, and root development were significantly enriched by upregulated DEGs in the comparison of RB vs RBG, while GO terms related to leaf and root development and plant hormone responses were significantly enriched by downregulated DEGs. The comparison between RB and RBW showed that the GO terms related to response to light stimulus/UV-B, photosynthesis, and regulation of stomatal closure were significantly enriched by upregulated DEGs, while processes of multicellular organism growth, response to oxidative stress, regulation of chlorophyll biosynthesis, basipetal auxin transport, and negative regulation of abscisic acid biosynthesis were significantly enriched by downregulated DEGs. In the comparison between RB and RBY, carbohydrate metabolic process was significantly enriched by upregulated DEGs, while regulation of cell development, leaf development and cell proliferation were significantly enriched by downregulated DEGs. In addition, in the comparison of W with RB, the BP of auxin polar transport was significantly enriched by upregulated DEGs, and the processes of regulation of cell growth and leaf development were significantly enriched by downregulated DEGs.

### Significant pathway enrichment analysis of DEGs

**Fig. 6:**
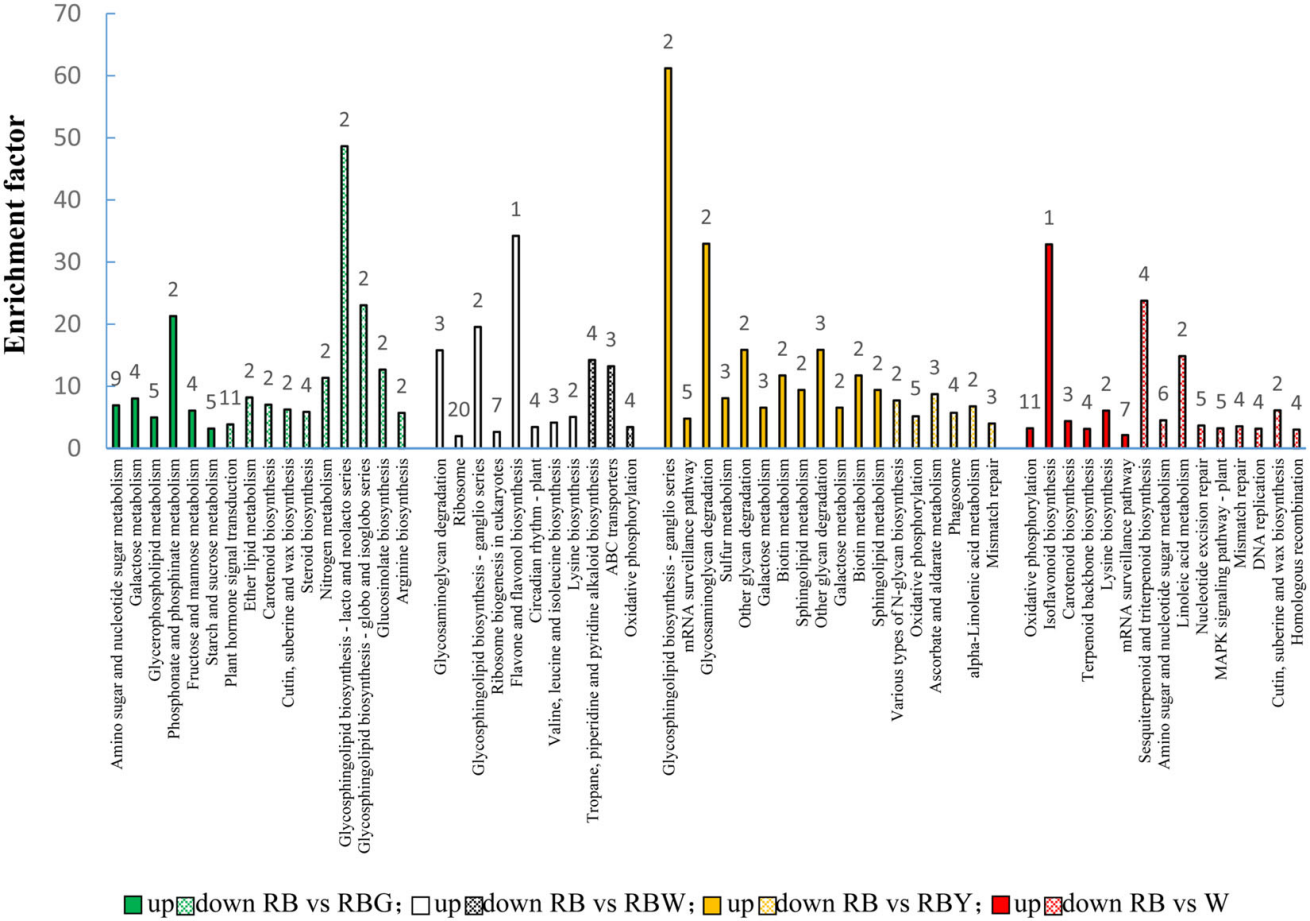
Significantly enriched KEGG pathways of differentially expressed genes (DEGs) in pairwise comparisons at the *P* < 0.05 level. The enrichment factor represents the ratio of the proportion of genes annotated to the pathway among DEGs to the proportion of genes annotated to the pathway among all genes. The number at the top of the vertical bars represents the number of DEGs enriched in the corresponding pathway. RB red–blue light, RBG red–blue–green light, RBW red–blue–white light, RBY red–blue–yellow light, W white light

For an exploration of the biological functions of the DEGs, the pathways significantly enriched by DEGs were identified using KEGG analyses (Fig. 6), and the DEGs involved in these pathways are listed (Supplementary Table S4). In the RB vs. RBG comparison, the pathways of carbohydrate metabolism (amino sugar, nucleotide sugar, galactose, fructose, mannose, starch, and sucrose) were significantly upregulated by total DEGs and exclusive DEGs, while pathways such as plant hormone signal transduction, glycosphingolipid biosynthesis, nitrogen metabolism, glucosinolate biosynthesis, and arginine biosynthesis were significantly downregulated (Fig. 6 and Supplementary Fig. S4). In the plant hormone signal transduction pathway, auxin-responsive protein IAA (AUX/IAA) was coregulated by nine DEGs and SAUR family protein (SAUR) was downregulated by three DEGs under supplemental G. In the cytokinin and jasmonic acid signal pathway, the two-component response regulator family A-ARR and the jasmonate ZIM domain-containing protein JAZ were upregulated by *BnaC05g14720D* and *BnaC03g71460D*, respectively (Supplementary Fig. S5a and Supplementary Table S4).

In the RB vs. RBW comparison, a total of three pathways, including oxidative phosphorylation, were significantly downregulated, and four DEGs were significantly involved in the downregulation of the oxidative phosphorylation pathway (Fig. 6 and Supplementary Table S4). In addition, ascorbate and aldarate metabolism was downregulated by two exclusive DEGs, while the plant hormone signal transduction pathway was significantly upregulated by exclusive DEGs under supplemental W (Supplementary Fig. S4). The auxin-responsive GH3 gene family (GH3), SAUR, and protein phosphatase 2C (PP2C) were all upregulated by DEGs (Supplementary Fig. S7b). *PsaD* and *Lchb1/2*, which are related to photosynthesis and photosynthesis-antenna proteins, were downregulated and upregulated under supplemental W, respectively (Supplementary Fig. S6a, b).

In the RB vs. RBY comparison, the DEGs significantly upregulated pathways such as glycosphingolipid metabolism, galactose metabolism, and other glycan degradation, and significantly downregulated the pathways of oxidative phosphorylation, ascorbate, and aldarate metabolism, phagosome, and mismatch repair (Fig. 6 and Supplementary Table S4). In addition, the KEGG pathways related to photosynthesis were downregulated, and the photosynthesis-responsive gene *PsbQ* in PSII was downregulated under supplemental Y (Supplementary Fig. S6a). In the cytokinin and jasmonic acid signal pathways, the two-component response regulator A-ARR family and JAZ were upregulated (Supplementary Fig. S5c).

In the RB vs. W comparison, the upregulated DEGs were significantly enriched in pathways such as oxidative phosphorylation and isoflavonoid and Car biosynthesis, while the downregulated DEGs were significantly enriched in the pathways of sesquiterpenoid and triterpenoid biosynthesis; amino sugar and nucleotide sugar metabolism; cutin, suberine, and wax biosynthesis; and the repair of nucleotide excision and mismatch (Fig. 6 and Supplementary Fig. S4). *Lhcb1*, a photosynthesis-responsive gene, was downregulated under sole W treatment (Supplementary Fig. S6b), and the peroxisomal response genes of the KEGG pathways, namely, *CAT*, *FAR*, and *ACSL* were also downregulated (Supplementary Fig. S7).

## Discussion

### Effects of supplemental light and light cross-talk

The regulatory effects of different lights on rapeseed seedlings at the transcriptome level were investigated by RNA-Seq analysis. Through pairwise comparison with RB, the number of DEGs induced by sole W or supplemental W was noticeably more than that induced by supplemental Y or G (Fig. 5a), suggesting that the broad spectra and/or cross-talk between different wavelengths might regulate DEGs in rapeseed. Comparison of expression patterns revealed that two genes (*BnaC01g26130D* and *BnaA05g31530D*) had significantly upregulated expression and overlapped RB vs. RBG and RB vs. W/RBW, and 66 overlapping DEGs showed similar expression in the comparison of RB vs. RBY and RB vs RBW (Fig. 5a), suggesting that Y is more likely substituted by light of other wavelengths to achieve certain effects similar to those of supplemental Y, while G-induced DEGs showed more distinctive involvement in the morphological and physiological alteration of rapeseed seedlings. In addition, eleven DEGs overlapped between RB vs. RBG and RB vs. RBW, and ten genes had similar expression patterns (Supplementary Table S1). These DEGs functioned in the hormone signaling pathway and response to light stimulus, suggesting that these genes possibly contributed to the satisfactory growth of rapeseed seedlings under supplemental G/W.

### Green light supplementation improves the growth and photosynthesis of rapeseed seedlings

Supplemental green light enhanced the growth of rapeseed seedlings. Similar results were obtained in other species, such as cucumber^24^ and lettuce^27^, under green light supplementation. In this study, supplemental W and G exhibited similar morphological alterations (Fig. 1a, b). Given the large proportion of green light in W, we speculate that green light plays a role in this process (Supplementary Fig. S8). In addition, the GO analyses showed that the DEGs induced by supplemental G enriched the GO terms related to root hair cell, root system, and lateral root development, which were not enriched by other light treatments (Fig. 5d and Supplementary Table S3), suggesting that the G-induced genes play an important role in root growth and activity. Simple leaf morphogenesis and negative regulation of leaf senescence were significantly downregulated by supplemental G, and simple leaf morphogenesis as well as multicellular organism growth was also significantly downregulated by supplemental W (Supplementary Table S3). In contrast, leaves under supplemental G/W developed better, characterized by three layers of palisade cells and lower specific leaf area, than those under sole W and supplemental Y, which led to an increase in Pn (Figs. 2 and 3a). The higher Pn under supplemental G and W, where the blue/green ratio was changed compared to that under RB, might also be related to cryptochrome activity alteration. As sensors of the blue/green ratio of natural radiation^31^, cryptochromes regulate the biosynthesis of many photosynthetic proteins and enzymes^32^. The current results also showed that the DEGs under supplemental W were significantly enriched in the MFs related to photoreceptor activity (Fig. 5e). Under the supplemental W treatment, phytochromes might also be involved in photosynthetic regulation due to the alteration of the red/far-red ratio^33^. In addition, the DEGs under supplemental G were significantly enriched in BPs related to plant hormone signal transduction, including the auxin, cytokinin, and jasmonic acid pathways (Fig. 6, Supplementary Fig. S5, and Supplementary Table S3), and thus regulated cell enlargement and division, shoot initiation, and plant growth and senescence^34^. In addition, the exclusively upregulated DEGs under supplemental W were significantly enriched in BPs related to plant hormone signal transduction (Supplementary Fig. S4). Therefore, better growth of rapeseed seedlings under supplemental G is related to green light-induced DEGs activating plant hormone signaling pathways and thus causing a series of cascade responses, while growth and photosynthetic responses of the supplemental W are partly attributed to green light, although the pathways and processes regulated by these DEGs differ from those under supplemental G. The combined effects of multiple processes ultimately led to similar photosynthesis and growth of plants under supplemental W and G.

### Yellow light supplementation alters leaf morphology and restrains photosynthesis

Supplemental Y significantly changed leaf development at the leaf cross-sectional level (Fig. 2). Unexpectedly, the number of palisade cell layers of leaves grown under supplemental Y as well as those grown under sole W was actually reduced to two layers (Fig. 2a). However, a decreasing palisade cell layer was previously observed in plant leaves grown under monochromatic light or low light^35,36^. Further comparison between the supplemental Y and sole W treatments showed that most of the growth and leaf traits, as well as chlorophyll fluorescence parameters and oxidative damage, had similar variations (Figs. 1–4 and Supplementary Fig. S1). Therefore, given the large proportion of yellow light in W, we presumed that these traits in the sole W treatment were induced by yellow light (Supplementary Fig. S8). Transcriptomic results showed that supplemental Y and sole W significantly downregulated leaf development and cell growth and proliferation (Supplementary Table S3), implying that leaf architecture development under supplemental Y and sole W was inhibited by some of the DEGs that suppressed cell proliferation, division, and growth, resulting in differences in the leaves under Y and sole W compared to the other three treatments. Consequently, a lower Pn was observed under supplemental Y (Fig. 3a).

The reduced photosynthetic rate also caused a reduction in starch and NSC accumulation in leaves grown under supplemental Y (Fig. 3d, f). The result was consistent with the study of Chen et al.^37^, but the starch accumulation under supplemental Y reached levels observed under supplemental G/W after daytime (Supplementary Fig. S2a–c), suggesting that leaves grown under supplemental Y have a higher starch synthesis rate than those grown under other supplemental lights during the daytime. The GO enrichment analyses revealed that starch synthase activity was regulated by DEGs under supplemental Y (Fig. 5f) and thus might promote starch synthesis. The DEGs induced by sole W were enriched in the amylopectin biosynthetic process (Fig. 5g), which might have contributed to the highest starch accumulation observed under sole W. In contrast to the levels of starch after and before the daytime (Supplementary Fig. S2a–c), we knew that starch was decomposed/consumed much less under the supplemental G treatment than under the other treatments during the night-time. The KEGG enrichment analyses revealed that oxidative phosphorylation was upregulated by the exclusive DEGs under the supplemental Y and W treatments, but downregulated under the sole W treatment (Supplementary Fig. S4). In contrast, glycosaminoglycan degradation was significantly upregulated under supplemental Y and W, and other glycan degradation was significantly upregulated under supplemental Y. Under supplemental G, several enrichment pathways related to carbohydrate metabolism were significantly upregulated (Fig. 5 and Supplementary Table S4). On the basis of the above analysis, we can deduce that starch degradation in darkness is regulated by these DEGs in different ways and may be balanced by several pathways.

### Yellow light causes a low-light-like environment and induces the corresponding coping strategies of rapeseed seedlings

The lower Fv/Fm observed under supplemental Y and sole W implied that the plants were in a suboptimal environment (Fig. 3b). GO terms associated with the response to high light intensity were significantly enriched by the DEGs under supplemental G (Supplementary Table S3), and the DEGs under supplemental W were also associated with these GO terms (data not shown), suggesting that the plants grown under supplemental G/W might capture more light energy than those grown under supplemental Y and sole W. Moreover, high specific leaf areas, a typical trait of the shade avoidance response, were observed under both the supplemental Y and sole W treatments (Fig. 1), which was a result of leaf expansion for capturing more light^38^. In addition, PsbQ is required for photoautotrophic growth under low light conditions^39^, and *PsbQ* was downregulated under supplemental Y as well (Supplementary Fig. S6a). Therefore, we deduced that plants grown under supplemental Y as well as sole W may suffer from a reduction in light absorption and thus from a low-light-like environment.

Under these suboptimal environmental conditions, supplemental Y and sole W significantly increased MDA levels and membrane injury as well as free radical levels compared with the other treatments (Fig. 4a–d), indicating relative damage to the membrane system and ROS balance. Studies have shown that monochromatic RB cause oxidative stress and destroy the cell membrane^36^. Samuolienė et al.^25^ also showed partly similar results, where supplemental LED light enhanced the antioxidant capability of lettuce for protection from the photooxidative state. In the current study, higher O_2_^−^ and H_2_O_2_ levels under the supplemental Y and sole W conditions resulted in a high MDA content, and higher SOD, CAT, and POD activities were correspondingly required to remove ROS^40,41^. Analysis of the significantly enriched GO categories revealed that ascorbate metabolism (Supplementary Table S4) and CAT (Supplementary Fig. S7) were upregulated under sole W, which is consistent with the results shown in Fig. 4f, h. It has been reported that higher SOD and POD activities improve the recovery of photosynthesis in plants subjected to unfavorable environments^42^. Therefore, the higher Pn induced by sole W than by supplemental Y might contribute to a similar effect as that observed by Sales et al.^42^. In addition, AsA, as the main member of the non-enzymatic scavenging systems, presented a higher level in plants grown under sole W than in those grown under supplemental Y (Fig. 4h). The combination of the enzymatic and non-enzymatic radical scavenging system engenders a more marked protective effect against photoinhibition under sole W^43^. KEGG analysis showed that the synthesis and decomposition of secondary metabolites were also significantly enriched by DEGs under sole W, including sesquiterpene and chitinase biosynthetic processes (Fig. 6 and Supplementary Table S3). These substances can be used in the cell skeleton, are strong antioxidants, and have pharmacological activity^44,45^. Therefore, rapeseed grown under sole W had a higher capacity to quench ROS due to an efficient antioxidant system, and thus, the plants could maintain active photosynthesis^46^.

In summary, supplemental light added to RB improved dry mass accumulation to varying degrees and promoted the healthy growth of rapeseed seedlings. The effects of white light are closely related to its spectral distribution, and the spectrum of white LEDs needs to be optimized for plant cultivation in the future. Transcriptome analysis showed that supplemental G had a more distinctive effect on rapeseed seedlings and could enhance photosynthesis and then significantly improve the growth of rapeseed seedlings, and supplemental W had similar effects, which can mostly be attributed to the green wavelengths in white light. Supplemental Y significantly restrained leaf development at the leaf cross-sectional level and sharply decreased the photosynthetic rate, but regulated starch synthase activity and then promoted starch synthesis to supply a comparable level of carbohydrates as the RB control for seedling growth. Sole W caused effects similar to those of supplemental Y, which could be partly attributed to the yellow wavelengths in white light. Moreover, plants grown under sole W maintained a comparable level of the photosynthetic rate as the RB control due to a stronger ability to scavenge ROS. Therefore, supplementation of RB with green light is recommended for rapeseed seedling cultivation in a controlled environment.

## Materials and methods

### Plant materials and growth conditions

Seeds of rapeseed (*Brassica napus* L. cv. Njau 4) were germinated in an incubator at 28 °C after a 24-h soak in distilled water at 30 °C in the dark, and the germinated seeds were sown in a cultivation medium and grown under a white fluorescent lamp. After the emergence of the second true leaf, seedlings with roughly homogenous growth were transplanted to plastic pots (12-cm diameter). After one day of recovery, 150 plants were grown under five types of light, that is, RB (the control group), RBG, RBW, RBY, and sole W, provided by LEDs for 15 days at the same photosynthetic photon flux density (PPFD; 400 ± 10 μmol m^−2^ s^−1^), as measured by a quantum sensor (LI-250A, LI-COR, USA), and the PPFD ratio of R to B to supplemental light (G/Y/W) was 9:3:1. The spectral distributions of LEDs of different colors, as shown in Supplementary Fig. S8, were determined using a spectroradiometer (OPT-2000, ABDPE Co., Beijing, China). The peak wavelengths of R, B, G, and Y were 670, 455, 520, and 595 nm, respectively, and the full-width at half-maximum of all lights was 20 nm. All plants were exposed to a 12 h photoperiod (lighting from 08:30 to 20:30) with (60 ± 10) % relative humidity and a day/night temperature of (25 ± 2)/(17 ± 2) °C.

### Measurements of growth and physiological traits

After 15 days of irradiation, three plants per treatment were randomly selected to assay growth and physiological traits. The plant height, stem diameter, and DW were recorded, and the health index was determined using the following formula: (stem diameter/plant height + root/shoot DW) × plant DW. The specific leaf area of rapeseed was determined by the ratio of fresh leaf area to leaf DW. Root activity was measured using the method adopted by Li et al.^47^. The third fully expanded leaf from the top was selected for leaf sectioning, and the anatomical structure and stomata of the leaf were viewed under a DP71 optical microscope (Olympus Inc., Tokyo, Japan) using the method of Li et al.^47^. Leaf compactness was the ratio of palisade tissue to leaf thickness. Aperture length, width, and area were measured for at least 32 stomata selected randomly. The number of stomata per field of view in the leaf epidermis was recorded to calculate the stomatal frequency.

The Pn of the third fully expanded leaf was measured using a LI-6400 portable photosynthesis measurement system (LI-COR, Lincoln, NE, USA). Chlorophyll fluorescence parameters (Fv/Fm, qN, Y(II), Y(NPQ), Y(NO), and rETR) were determined with a fluorometer (PAM2100, Walz, Germany) according to a previous method^7^. The levels of carbohydrates, including soluble sugar, starch, and sucrose, were determined as reported previously^48,49^, and leaf discs were stained with an iodine solution to visualize starch in leaves^50^.

The MDA content was determined using a thiobarbituric acid solution according to Heath and Packer^51^ and calculated by using a molar extinction coefficient of 155 mM^−1^ cm^−1^. The degree of membrane lipid peroxidation was used to evaluate membrane integrity, and the membrane injury index and ROS levels were determined using the method of Jahan et al.^52^. For the measurement of the antioxidants, SOD, POD, and CAT activities and the radical scavengers AsA and Car were quantified by previously described procedures^41,42,49^.

### RNA isolation and transcriptome sequencing

After 15 days of irradiation, leaf samples of three plants per treatment were collected and ground into powder in liquid nitrogen, and then, total RNA was extracted using a Plant RNA Kit (TIANGEN Technology, Co., Ltd., Beijing, China). RNA concentration was measured using an RNA Assay Kit with a Qubit Fluorometer (Life Technologies, CA, USA), and RNA integrity was assessed with a Bioanalyzer 2100 (Agilent Technologies, CA, USA). Then, mRNA was enriched from total RNA using poly-T oligo-attached magnetic beads, and the mRNA molecules were fragmented and subsequently used in first- and second-strand complementary (cDNA) syntheses. The cDNA was subsequently subjected to end repair and poly (A) and unique adapter ligation. Before sequencing, the cDNA fragments were amplified and purified. RNA purity was checked using a spectrophotometer (IMPLEN, CA, USA). The purified amplification products were sequenced on a Novaseq-PE 150 platform. The original sequencing data were defined as raw reads. The clean reads were generated from the raw reads after removing the low-quality reads, mismatches, and adaptor sequences. The reference genome *Brassica_napus*_v4.1 (http://www.genoscope.cns.fr/brassicanapus/data/) was used. At the same time, the Q20, Q30, GC content, and sequence duplication level of the clean data were calculated. All downstream analyses were based on clean data with high quality.

### RNA-Seq data analysis

Differential gene expression analysis of four comparisons was performed using the DESeq R package (1.10.1). For RNA-Seq data analysis, the resulting *P* values were adjusted using the Benjamini–Hochberg approach for controlling the false discovery rate (FDR). The DEGs were obtained using |log 2 (fold change)| ≥ 1 and FDR < 0.05 as screening criteria. The DEGs were subjected to GO analyses, and all DEGs were mapped to GO terms in the database (http://www.geneontology.org/). To analyze the pathways that were significantly associated with DEGs, we used the same method to blast the DEGs against the KEGG database (https://www.kegg.jp/). The gene numbers of each term/pathway were calculated, and the hypergeometric test was used to analyze the significantly enriched pathways.

### Quantitative real-time polymerase chain reaction

Total RNA was extracted from each sample by using TRIzol reagent (Invitrogen, CA, USA), and ~0.5 μg of total RNA was used for cDNA synthesis using HiScript® II Q RT SuperMix for qPCR (+gDNA wiper) (Vazyme, Nanjing, China). After diluting the cDNA reaction mixture five times, 1 μL of the reaction mixture was used as template in a 10-μL reaction system. In addition, the reaction system contained 0.4 μL of 10 μmol L^−1^ gene-specific primers (Supplementary Table S5) and 5 μL of ABI (Shanghai, China) SYBR® Select Master (2×). qRT-PCR was performed on an Eppendorf real-time PCR machine (Hamburg, Germany). A rapeseed actin gene, *actin-2*, was used as the reference gene for normalization. All experiments involved three biological replicates and three technical replicates.

## Supplementary information

suppplemental figures

supplemental tables
