# Supplementary material for: RNA-Seq analysis reveals the growth and photosynthetic responses of rapeseed (Brassica napus L.) under red and blue LEDs with supplemental yellow, green, or white light
Source: Hortic Res. 2020 Dec 1;7:206. doi: 10.1038/s41438-020-00429-3 (PMC7705657; doi:10.1038/s41438-020-00429-3)
Supplement: Supplementary file 1 — suppplemental figures [file 41438_2020_429_MOESM1_ESM.pdf]

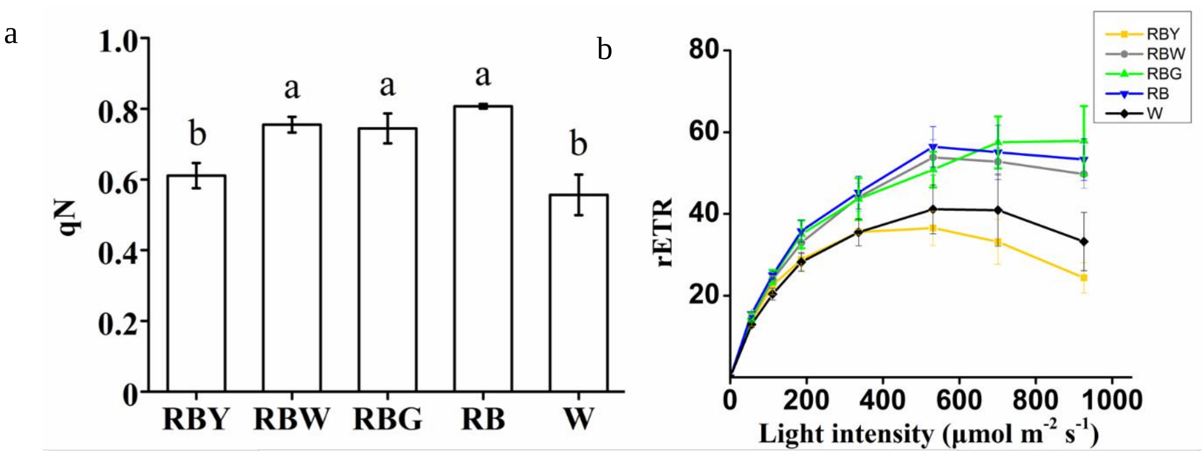

Fig. S1 Chlorophyll fluorescence parameter including qN (a) and rETR (b) in the top third leaf of rape under different light treatments. RB, red-blue light; RBG, red-blue-green light; RBW, red-blue-white light; RBY, red-blue-yellow light; W, white light. Vertical bars are means ± SD (n=3). Bars labelled with lowercase letters are significantly different at $P < 0.05$ level.

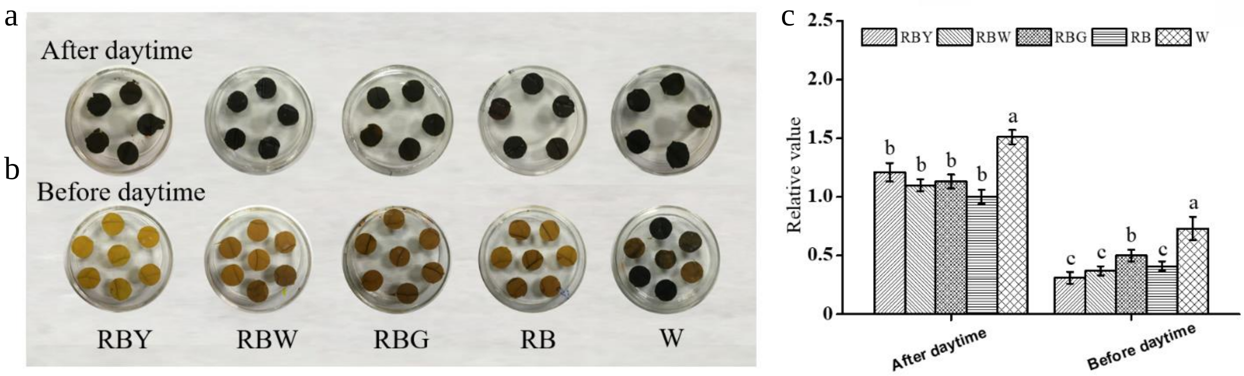

Fig. S2 The leaf discs stained with iodine solution after daytime and before daytime respectively (a and b) and the relative content of starch (c) in different light treatments. RB, red-blue light; RBG, red-blue-green light; RBW, red-blue-white light; RBY, red-blue-yellow light; W, white light. Vertical bars are means ± SD (n=5). Bars labelled with lowercase letters are significantly different at $P <$ 0.05 level.

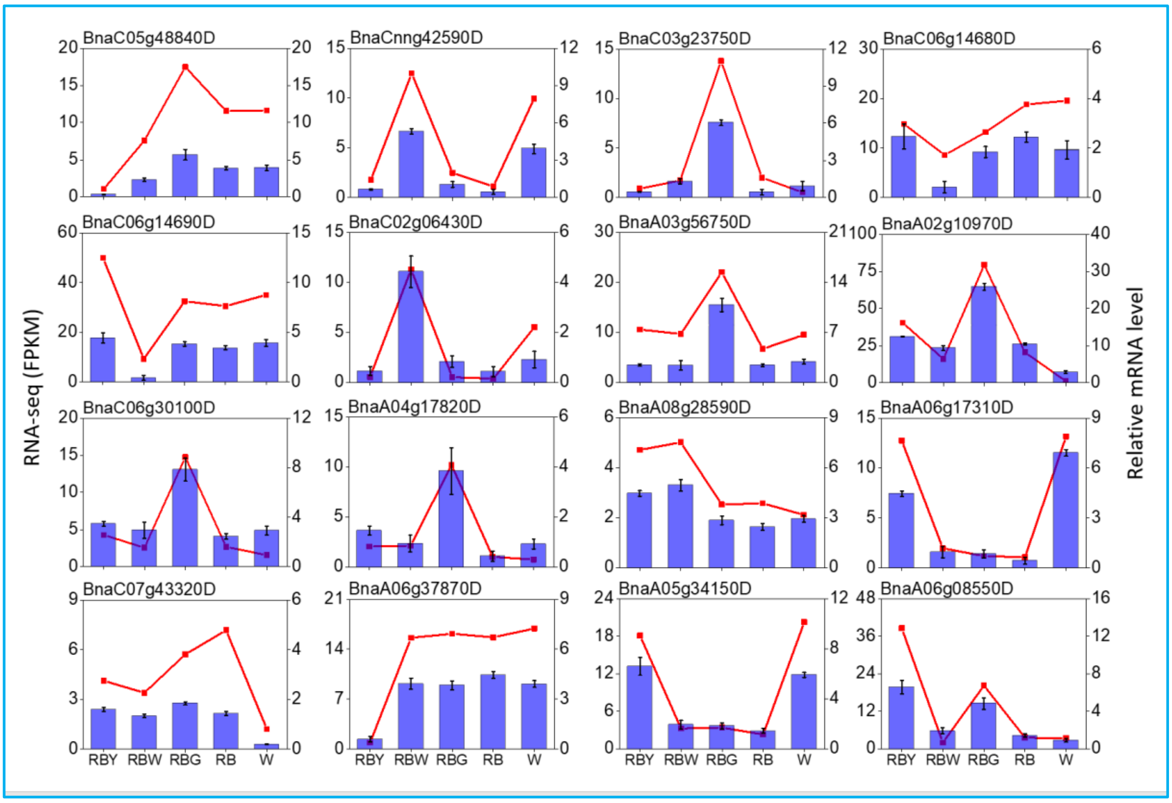

Fig. S3 Validation of transcript abundance obtained from RNA-seq using qRT-PCR. RB, red-blue light; RBG, red-blue-green light; RBW, red-blue-white light; RBY, red-blue-yellow light; W, white light. Sixteen randomly chosen genes were used for validation. Red line and blue column graph indicate relative expression by RNA-seq and qRT-PCR under different light treatments, respectively. Vertical bars are means ± SD (n=3). Bars labelled with lowercase letters are significantly different at *P* < 0.05.

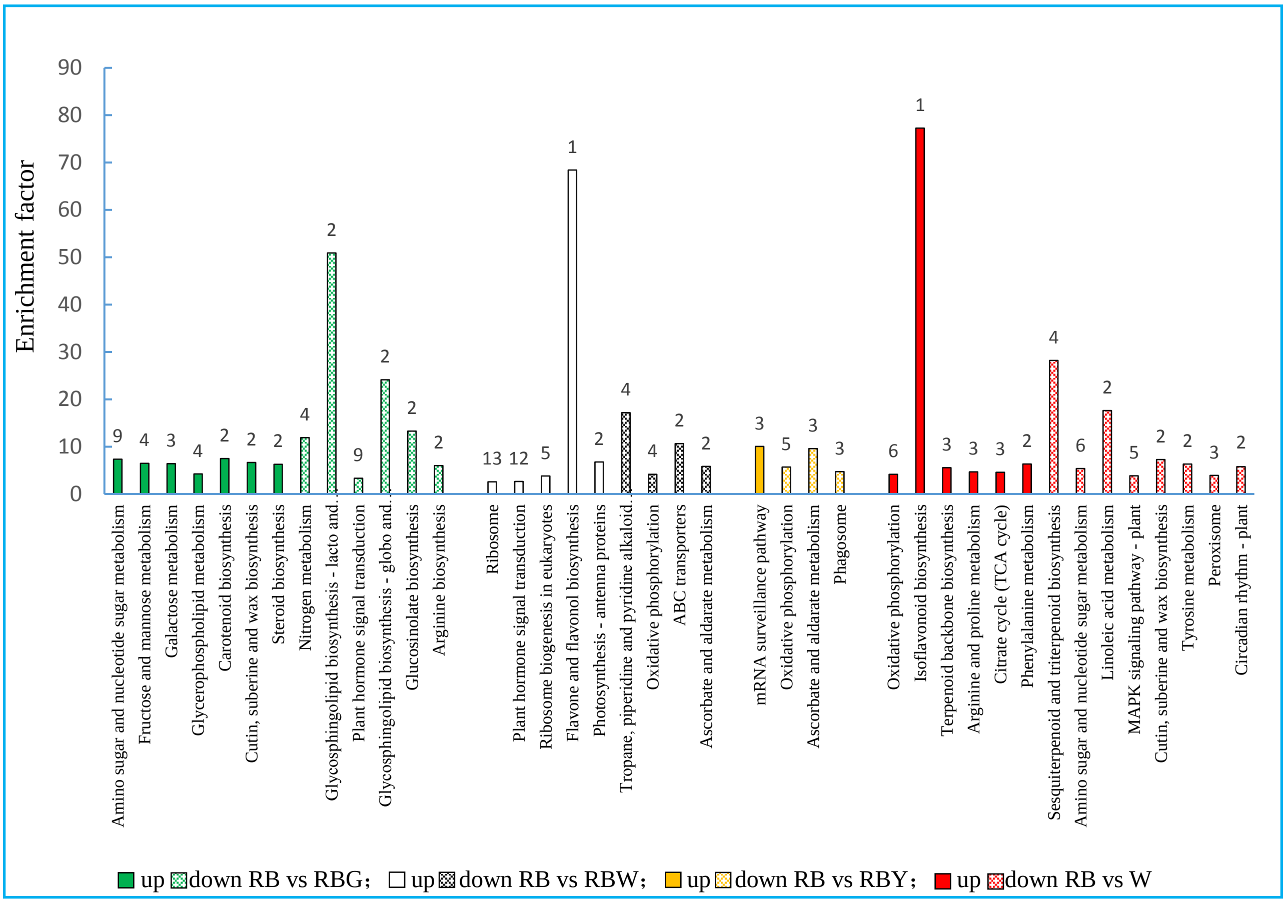

Fig. S4 Functional categories of exclusive differentially expressed genes (DEGs) based on KEGG enrichment analyses. Significantly enriched KEGG categories were analyzed in pairwise comparisons (RB vs RBG, RB vs RBW, RB vs RBY and RB vs W) at $P < 0.05$ level. Enrichment factor represents the ratio of the proportion of genes annotated to the pathway among DEGs to the proportion of genes annotated to the pathway among all genes. The digit in the top of column graph represents the number of DEGs enriched in corresponding pathway. RB, red-blue light; RBG, red-blue-green light; RBW, red-blue-white light; RBY, red-blue-yellow light; W, white light.

a

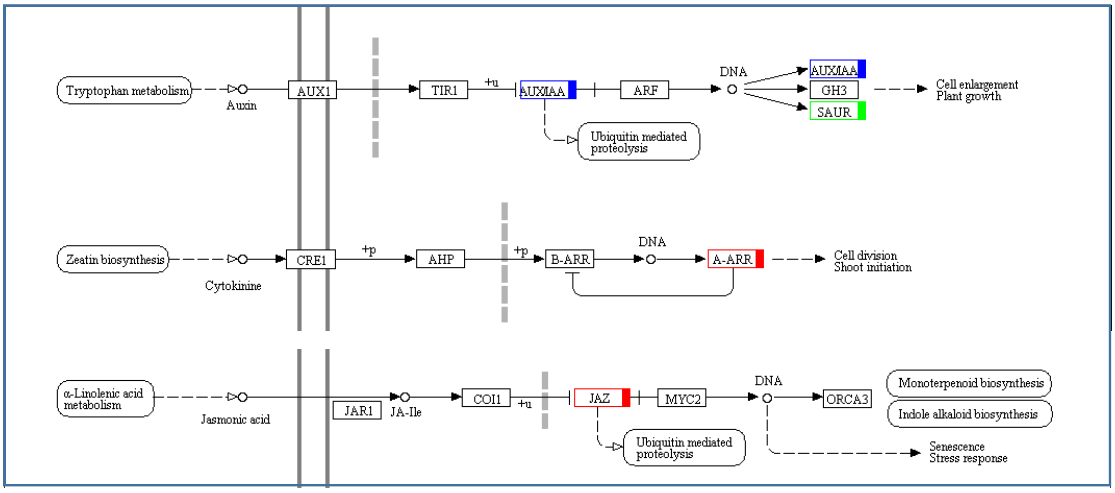
Tryptophan metabolism
Auxin
AUX1
TIR1
+u
AUX/IAA
ARF
DNA
AUX/IAA
GH3
SAUR
Cell enlargement
Plant growth
Ubiquitin mediated proteolysis
Zeatin biosynthesis
Cytokinine
CRE1
+p
AHP
+p
B-ARR
DNA
A-ARR
Cell division
Shoot initiation
α-Linolenic acid metabolism
Jasmonic acid
JAR1
JA-Ile
COI1
+u
JAZ
MYC2
DNA
ORCA3
Monoterpenoid biosynthesis
Indole alkaloid biosynthesis
Ubiquitin mediated proteolysis
Senescence
Stress response

b

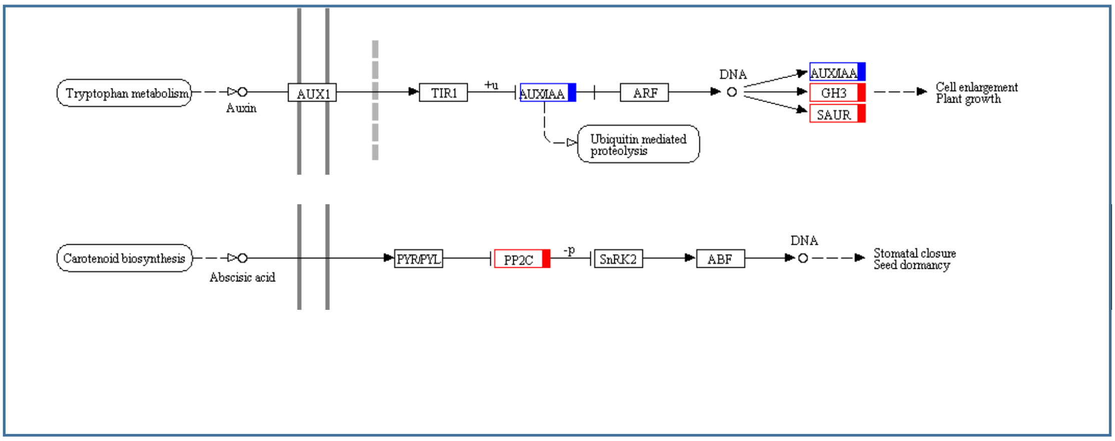
Tryptophan metabolism
Auxin
AUX1
TIR1
+u
AUX/IAA
ARF
DNA
AUX/IAA
GH3
SAUR
Cell enlargement
Plant growth
Ubiquitin mediated proteolysis
Carotenoid biosynthesis
Abscisic acid
PYR/PYL
PP2C
-p
SnRK2
ABF
DNA
Stomatal closure
Seed dormancy

c

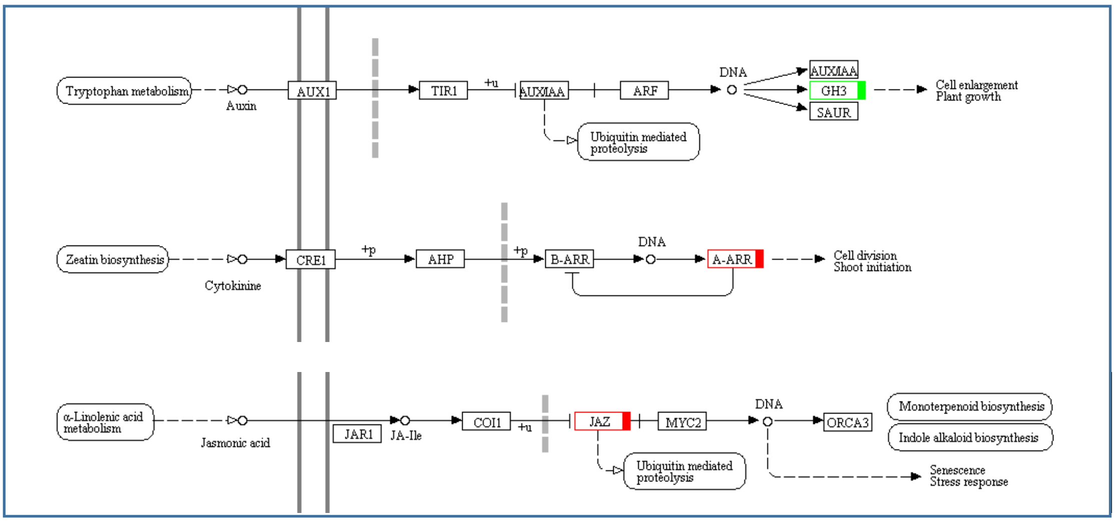
Tryptophan metabolism
Auxin
AUX1
TIR1
+u
AUX/IAA
ARF
DNA
AUX/IAA
GH3
SAUR
Cell enlargement
Plant growth
Ubiquitin mediated proteolysis
Zeatin biosynthesis
Cytokinine
CRE1
+p
AHP
+p
B-ARR
DNA
A-ARR
Cell division
Shoot initiation
α-Linolenic acid metabolism
Jasmonic acid
JAR1
JA-Ile
COI1
+u
JAZ
MYC2
DNA
ORCA3
Monoterpenoid biosynthesis
Indole alkaloid biosynthesis
Ubiquitin mediated proteolysis
Senescence
Stress response

d

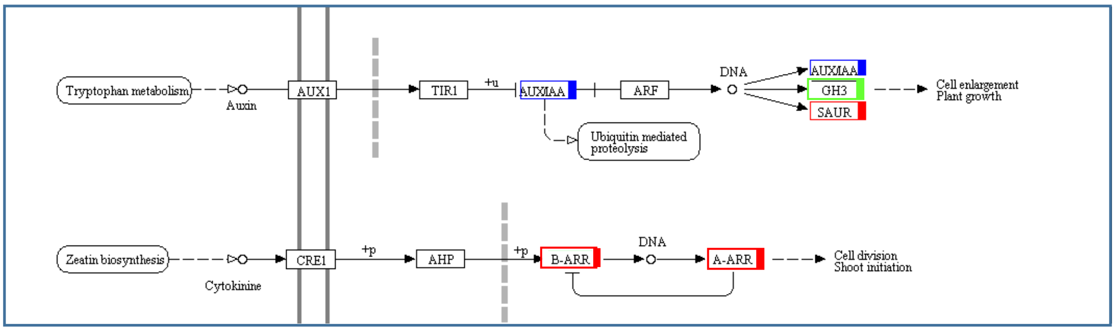

Fig. S5 Differentially expressed transcripts involved in plant hormone signal transduction. (a) RB vs RBG, (b) RB vs RBW, (c) RB vs RBY, (d) RB vs W. The red, green and blue separately represent up-regulation, down-regulation and up/down-regulation of the gene annotated to the enzyme. RB, red-blue light; RBG, red-blue-green light; RBW, red-blue-white light; RBY, red-blue-yellow light; W, white light.

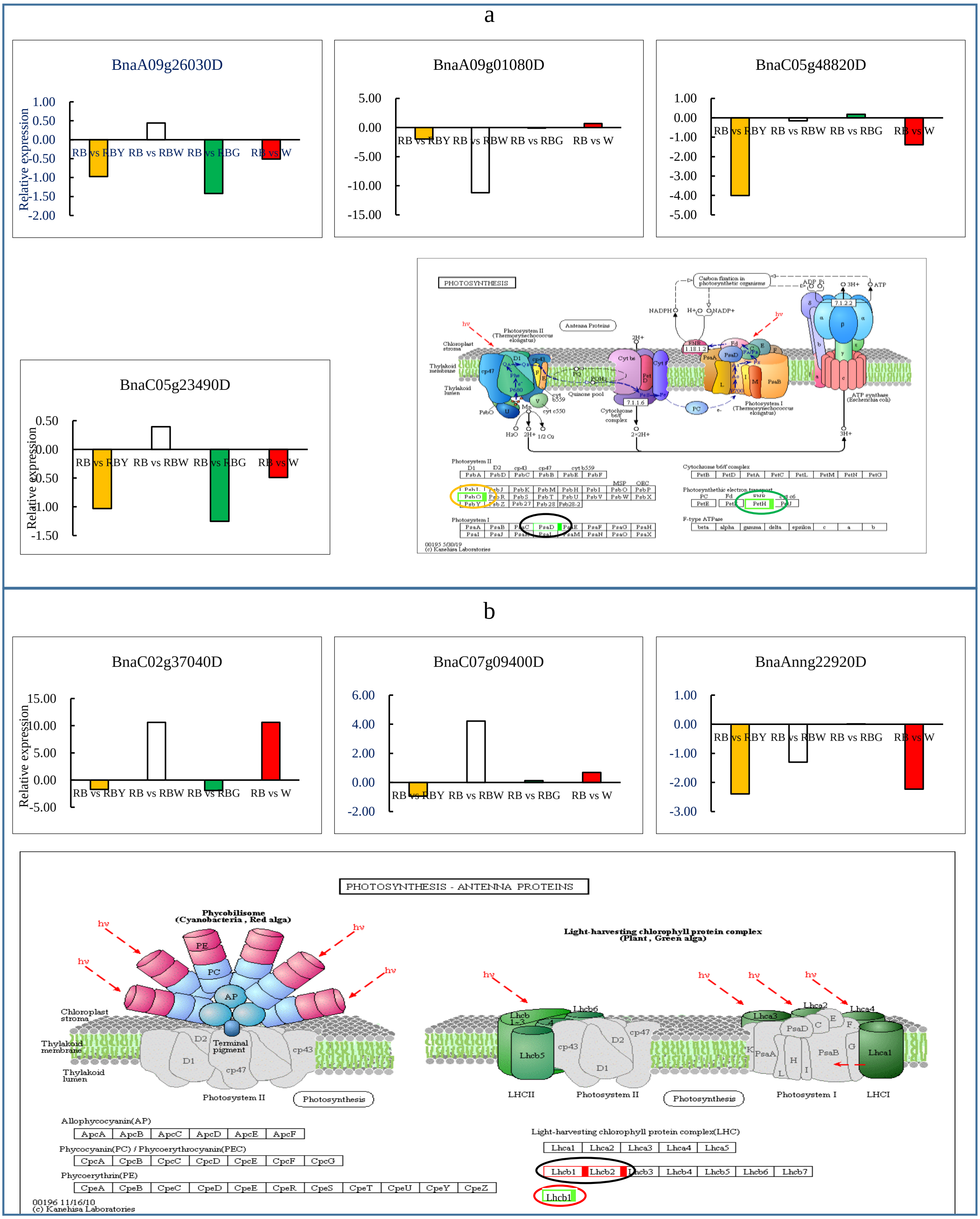

Fig. S6 Differentially expressed transcripts involved in photosynthesis and photosynthesis-antenna proteins. (a) photosynthesis, (b) photosynthesis-antenna proteins. The red, green and blue separately represent up-regulation, down-regulation and up/down-regulation of the gene annotated to the enzyme. The location circled by , , and represents responsive genes in the comparison of RB vs RBY, RB vs RBW, RB vs RBG and RB vs W, respectively. RB, red-blue light; RBG, red-blue-green light; RBW, red-blue-white light; RBY, red-blue-yellow light; W, white light.

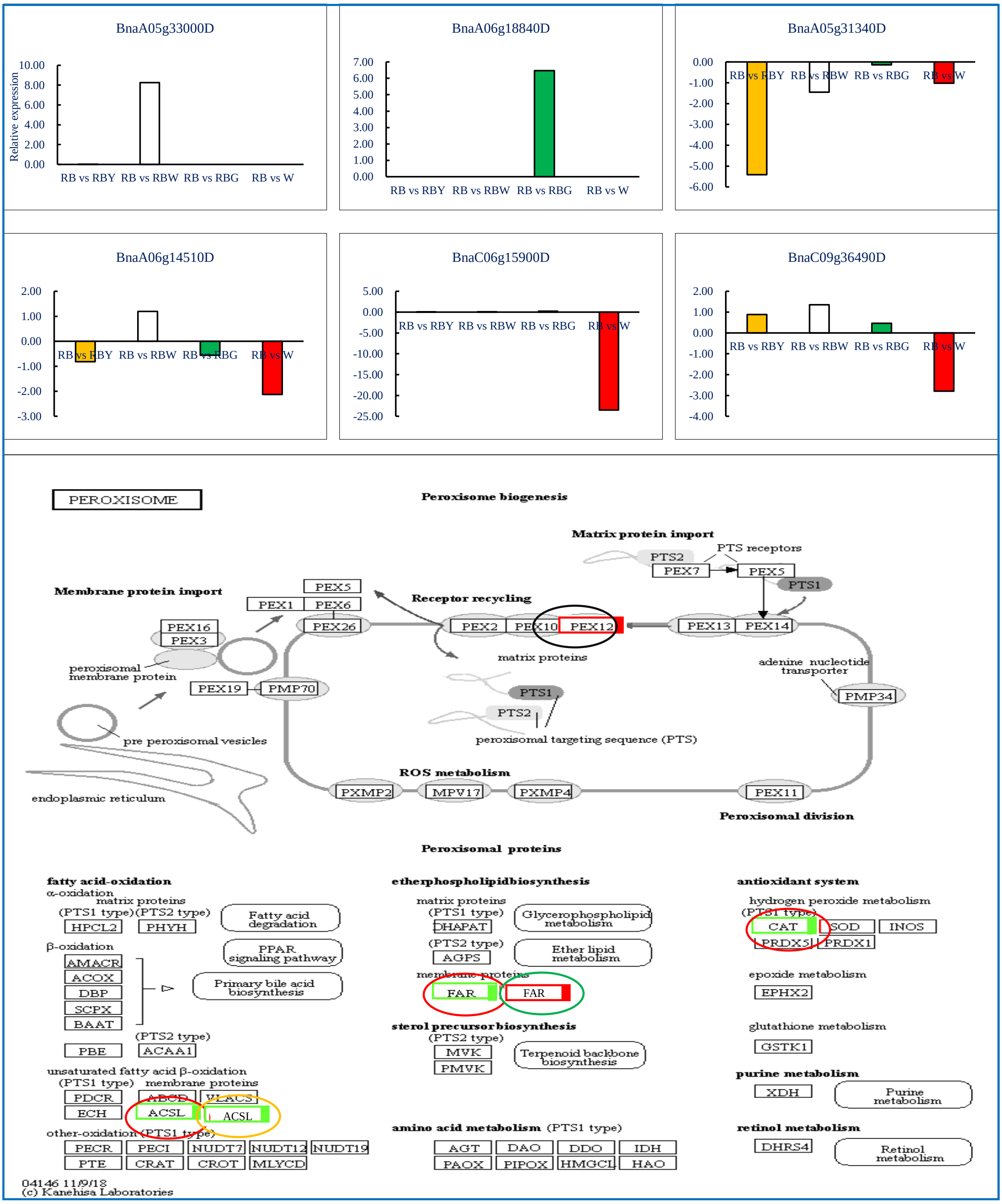

Fig. S7 Differentially expressed transcripts involved in peroxisome. The red, green and blue separately represent up-regulation, down-regulation and up/down-regulation of the gene annotated to the enzyme. The location circled by , , and represent responsive genes in the comparison of RB vs RBY, RB vs RBW, RB vs RBG and RB vs W, respectively. RB, red-blue light; RBG, red-blue-green light; RBW, red-blue-white light; RBY, red-blue-yellow light; W, white light.

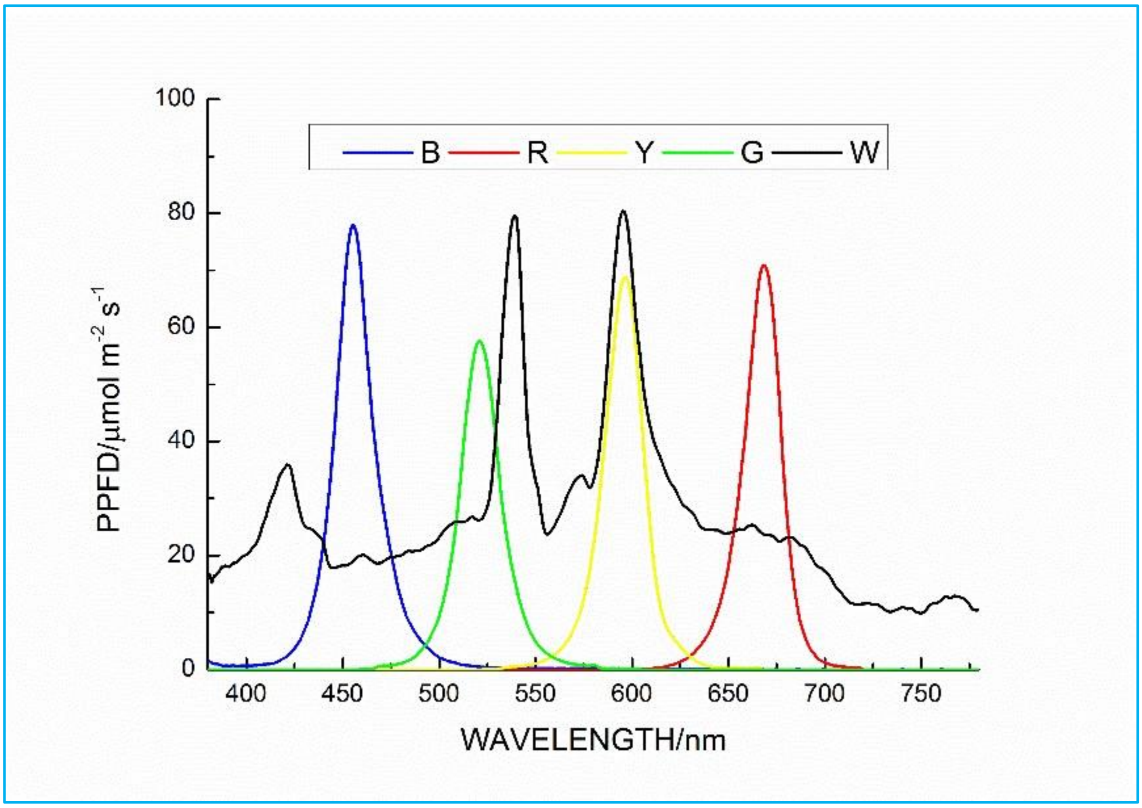

Fig. S8 The spectral distribution of red, blue, yellow, green and white lights. PPFD, photosynthetic photon flux density; B, blue light; R, red light; Y, yellow light; G, green light; W, white light.
