# Supplementary material for: RNA-Seq analysis reveals the growth and photosynthetic responses of rapeseed (Brassica napus L.) under red and blue LEDs with supplemental yellow, green, or white light
Source: Hortic Res. 2020 Dec 1;7:206. doi: 10.1038/s41438-020-00429-3 (PMC7705657; doi:10.1038/s41438-020-00429-3)
Supplement: Supplementary file 2 — supplemental tables [file 41438_2020_429_MOESM2_ESM.docx]

**Table S1 The common DEGs and their functions in the comparisons between RB vs RBG and RB vs RBW.** RB, red-blue light; RBG, red-blue-green light; RBW, red-blue-white light; RBY, red-blue-yellow light; W, white light.

| Gene ID | Regulated | | Go annotation / other annotation |
| --- | --- | --- | --- |
|  | RB vs RBG | RB vs RBW |  |
| BnaC01g06240D | down | down | DNA-binding transcription factor activity (GO:0003700); nucleus (GO:0005634); transcription, DNA-templated (GO:0006351); response to auxin (GO:0009733); auxin-activated signaling pathway (GO:0009734); response to red light (GO:0010114); response to far red light (GO:0010218). |
| BnaA01g26030D | down | down | DNA binding (GO:0003677); DNA-binding transcription factor activity (GO:0003700); nucleus (GO:0005634); transcription, DNA-templated (GO:0006351); response to water deprivation (GO:0009414); response to abscisic acid (GO:0009737); abscisic acid-activated signaling pathway (GO:0009738); embryo development ending in seed dormancy (GO:0009793);response to chitin (GO:0010200); hyperosmotic salinity response (GO:0042538); sequence-specific DNA binding (GO:0043565); transcription regulatory region DNA binding (GO:0044212); negative regulation of transcription, DNA-templated (GO:0045892); metal ion binding (GO:0046872). |
| BnaAnng15250D | down | down | nitrogen compound metabolic process (GO:0006807); simple leaf morphogenesis (GO:0060776). |
| BnaC01g26130D | up | up | - / WSTF, HB1, Itc1p, MBD9 motif 1 (Pfam annotation). |
| BnaA04g23610D | down | down | plasma membrane (GO:0005886); L-glutamine transmembrane transporter activity (GO:0015186); integral component of membrane (GO:0016021); amino acid export across plasma membrane (GO:0044746); seed development (GO:0048316). |
| BnaA09g38130D | down | down | - / F-box domain; Leucine Rich Repeat (Pfam annotation). |
| BnaC03g39020D | up | down | - / dehydrogenase/reductase SDR family member 7-like isoform X2 [Brassica napus] (nr annotation). |
| BnaA05g31530D | up | up | extracellular region (GO:0005576); killing of cells of other organism (GO:0031640); defense response to fungus (GO:0050832); |
| BnaC04g40810D | down | down | protein serine/threonine phosphatase activity (GO:0004722); nucleus (GO:0005634); plastid (GO:0009536);; response to wounding (GO:0009611); response to fungus (GO:0009620); abscisic acid-activated signaling pathway (GO:0009738); metal ion binding (GO:0046872); defense response to fungus (GO:0050832). |
| BnaAnng02700D | up | up | extracellular region (GO:0005576); killing of cells of other organism (GO:0031640); defense response to fungus (GO:0050832). |
| BnaA03g00870D | up | up | cytoplasm (GO:0005737); plasma membrane (GO:0005886); metal ion transport (GO:0030001); transition metal ion binding (GO:0046914); cellular transition metal ion homeostasis (GO:0046916). |

**Table S2** **The significantly enriched top 20 GO term and corresponding ID of the differentially expressed genes (DEGs).** RB, red-blue light; RBG, red-blue-green light; RBW, red-blue-white light; RBY, red-blue-yellow light; W, white light.

| **Comparison** | **GO ID** | **Term** |
| --- | --- | --- |
| **RB vs RBG** | GO:0060776 | simple leaf morphogenesis |
|  | GO:0009409 | response to cold |
|  | GO:0009631 | cold acclimation |
|  | GO:1900056 | negative regulation of leaf senescence |
|  | GO:0010200 | response to chitin |
|  | GO:0009873 | ethylene-activated signaling pathway |
|  | GO:0006807 | nitrogen compound metabolic process |
|  | GO:0009737 | response to abscisic acid |
|  | GO:0032880 | regulation of protein localization |
|  | GO:0009414 | response to water deprivation |
|  | GO:0050832 | defense response to fungus |
|  | GO:0042546 | cell wall biogenesis |
|  | GO:0022622 | root system development |
|  | GO:0001678 | cellular glucose homeostasis |
|  | GO:0009734 | auxin-activated signaling pathway |
|  | GO:0010411 | xyloglucan metabolic process |
|  | GO:0044746 | amino acid export across plasma membrane |
|  | GO:0019354 | siroheme biosynthetic process |
|  | GO:0009236 | cobalamin biosynthetic process |
|  | GO:0009738 | abscisic acid-activated signaling pathway |
| **RB vs RBW** | GO:0009416 | response to light stimulus |
|  | GO:0009536 | plastid |
|  | GO:0018298 | protein-chromophore linkage |
|  | GO:0097237 | cellular response to toxic substance |
|  | GO:0009570 | chloroplast stroma |
|  | GO:0010117 | photoprotection |
|  | GO:0010224 | response to UV-B |
|  | GO:1901006 | ubiquinone-6 biosynthetic process |
|  | GO:0006412 | translation |
|  | GO:0009881 | photoreceptor activity |
|  | GO:0008301 | DNA binding, bending |
|  | GO:0003954 | NADH dehydrogenase activity |
|  | GO:0009941 | chloroplast envelope |
|  | GO:0000785 | chromatin |
|  | GO:0035264 | multicellular organism growth |
|  | GO:0009813 | flavonoid biosynthetic process |
|  | GO:0009269 | response to desiccation |
|  | GO:0009523 | photosystem II |
|  | GO:0000783 | nuclear telomere cap complex |
|  | GO:0080085 | signal recognition particle, chloroplast targeting |
| **RB vs RBY** | GO:1904215 | regulation of protein import into chloroplast stroma |
|  | GO:0006996 | organelle organization |
|  | GO:0097264 | self-proteolysis |
|  | GO:0090392 | sepal giant cell differentiation |
|  | GO:0015811 | L-cystine transport |
|  | GO:0010475 | galactose-1-phosphate guanylyltransferase (GDP) activity |
|  | GO:0080047 | GDP-L-galactose phosphorylase activity |
|  | GO:0034450 | ubiquitin-ubiquitin ligase activity |
|  | GO:0004198 | calcium-dependent cysteine-type endopeptidase activity |
|  | GO:0008106 | alcohol dehydrogenase (NADP+) activity |
|  | GO:0030145 | manganese ion binding |
|  | GO:0070401 | NADP+ binding |
|  | GO:0016229 | steroid dehydrogenase activity |
|  | GO:0004033 | aldo-keto reductase (NADP) activity |
|  | GO:0033201 | alpha-1,4-glucan synthase activity |
|  | GO:0004652 | polynucleotide adenylyltransferase activity |
|  | GO:0080046 | quercetin 4'-O-glucosyltransferase activity |
|  | GO:0009011 | starch synthase activity |
|  | GO:0004373 | glycogen (starch) synthase activity |
|  | GO:0016820 | ATPase activity, coupled to transmembrane movement of substances |
| **RB vs W** | GO:0051762 | sesquiterpene biosynthetic process |
|  | GO:0016106 | sesquiterpenoid biosynthetic process |
|  | GO:0008061 | chitin binding |
|  | GO:0004568 | chitinase activity |
|  | GO:1904215 | regulation of protein import into chloroplast stroma |
|  | GO:0006032 | chitin catabolic process |
|  | GO:0016998 | cell wall macromolecule catabolic process |
|  | GO:0000272 | polysaccharide catabolic process |
|  | GO:0097264 | self-proteolysis |
|  | GO:0090392 | epal giant cell differentiation |
|  | GO:0009501 | amyloplast |
|  | GO:0006996 | organelle organization |
|  | GO:0009060 | aerobic respiration |
|  | GO:0102878 | (+)-alpha-barbatene synthase activity |
|  | GO:0008836 | diaminopimelate decarboxylase activity |
|  | GO:0102883 | (+)-beta-chamigrene synthase activity |
|  | GO:0010021 | amylopectin biosynthetic process |
|  | GO:0031559 | oxidosqualene cyclase activity |
|  | GO:0005090 | Sar guanyl-nucleotide exchange factor activity |
|  | GO:0080016 | (-)-E-beta-caryophyllene synthase activity |

**Table S3 List of the focused enrichment GO terms in some biological processes.** Significantly enriched GO catephgories (*P*-value < 0.01) were analyzed in pairwise comparisons (RB vs RBG, RB vs RBW, RB vs RBY, and RB vs W). Enrichment factor represents the ratio of the proportion of genes annotated to the pathway among DEGs to the proportion of genes annotated to the pathway among all genes. RB, red-blue light; RBG, red-blue-green light; RBW, red-blue-white light; RBY, red-blue-yellow light; W, white light. ‘◎’ means GO term no/no significant enrichment.

| **ID** | **Term** | **Enrichment factor** | | | | | | | |
| --- | --- | --- | --- | --- | --- | --- | --- | --- | --- |
|  |  | **RB vs RBG** | | **RB vs RBW** | | **RB vs RBY** | | **RB vs W** | |
|  |  | **Up** | **down** | **up** | **down** | **up** | **down** | **up** | **down** |
| GO:0009737 | response to abscisic acid | 4.21 | ◎ | ◎ | ◎ | ◎ | ◎ | ◎ | ◎ |
| GO:0016118 | carotenoid catabolic process | 135.42 | ◎ | ◎ | ◎ | ◎ | ◎ | ◎ | ◎ |
| GO:0010150 | leaf senescence | 6.16 | ◎ | ◎ | ◎ | ◎ | ◎ | ◎ | ◎ |
| GO:0080147 | root hair cell development | 19.20 | ◎ | ◎ | ◎ | ◎ | ◎ | ◎ | ◎ |
| GO:0009644 | response to high light intensity | 7.25 | ◎ | ◎ | ◎ | ◎ | ◎ | ◎ | ◎ |
| GO:0060776 | simple leaf morphogenesis | ◎ | 132.20 | ◎ | ◎ | ◎ | ◎ | ◎ | ◎ |
| GO:1900056 | negative regulation of leaf senescence | ◎ | 31.70 | ◎ | ◎ | ◎ | ◎ | ◎ | ◎ |
| GO:0022622 | root system development | ◎ | 31.90 | ◎ | ◎ | ◎ | ◎ | ◎ | ◎ |
| GO:0009733 | response to auxin | ◎ | 4.60 | ◎ | ◎ | ◎ | ◎ | ◎ | ◎ |
| GO:0009734 | auxin-activated signaling pathway | ◎ | 4.20 | ◎ | ◎ | ◎ | ◎ | ◎ | ◎ |
| GO:2000012 | regulation of auxin polar transport | ◎ | 16.96 | ◎ | ◎ | ◎ | ◎ | ◎ | ◎ |
| GO:0048527 | lateral root development | ◎ | 8.30 | ◎ | ◎ | ◎ | ◎ | ◎ | ◎ |
| GO:0008284 | positive regulation of cell proliferation | ◎ | 11.74 | ◎ | ◎ | ◎ | ◎ | ◎ | ◎ |
| GO:0009738 | abscisic acid-activated signaling pathway | ◎ | 2.87 | ◎ | ◎ | ◎ | ◎ | ◎ | ◎ |
| GO:0040008 | regulation of growth | ◎ | 3.18 | ◎ | ◎ | ◎ | ◎ | ◎ | ◎ |
| GO:0009416 | response to light stimulus | ◎ | 3.29 | 3.93 | ◎ | ◎ | ◎ | ◎ | ◎ |
| GO:0009768 | photosynthesis, light harvesting in photosystem I | ◎ | ◎ | 8.00 | ◎ | ◎ | ◎ | ◎ | ◎ |
| GO:0090333 | regulation of stomatal closure | ◎ | ◎ | 7.23 | ◎ | ◎ | ◎ | ◎ | ◎ |
| GO:0010224 | response to UV-B | ◎ | ◎ | 3.31 | 5.67 | ◎ | ◎ | ◎ | ◎ |
| GO:0035264 | multicellular organism growth | ◎ | ◎ | ◎ | 175.40 | ◎ | ◎ | ◎ | ◎ |
| GO:0006979 | response to oxidative stress | ◎ | ◎ | ◎ | 4.35 | ◎ | ◎ | ◎ | ◎ |
| GO:0010380 | regulation of chlorophyll biosynthetic process | ◎ | ◎ | ◎ | 16.97 | ◎ | ◎ | ◎ | ◎ |
| GO:0010540 | basipetal auxin transport | ◎ | ◎ | ◎ | 14.4 | ◎ | ◎ | ◎ | ◎ |
| GO:0090359 | negative regulation of abscisic acid biosynthetic process | ◎ | ◎ | ◎ | 105.22 | ◎ | ◎ | ◎ | ◎ |
| GO:0005975 | carbohydrate metabolic process | ◎ | ◎ | ◎ | ◎ | 3.16 | ◎ | ◎ | ◎ |
| GO:0001558 | regulation of cell growth | ◎ | ◎ | ◎ | ◎ | ◎ | 38.23 | ◎ | 24.09 |
| GO:2000024 | regulation of leaf development | ◎ | ◎ | ◎ | ◎ | ◎ | 24.66 | ◎ | 15.55 |
| GO:0042127 | regulation of cell proliferation | ◎ | ◎ | ◎ | ◎ | ◎ | 10.10 | ◎ | ◎ |
| GO:0009926 | auxin polar transport | ◎ | ◎ | ◎ | ◎ | ◎ | ◎ | 5.25 | ◎ |
| GO:0010597 | green leaf volatile biosynthetic process | ◎ | ◎ | ◎ | ◎ | ◎ | ◎ | ◎ | 37.01 |

**Table S4 List of significantly enrichment pathways by DEGs and the identifier of DEGs**. Significantly enriched KEGG categories (*P*-value < 0.05 and Q-value ≤ 1) were analyzed in pairwise comparisons (RB vs RBG, RB vs RBW, RB vs RBY and RB vs W). RB, red-blue light; RBG, red-blue-green light; RBW, red-blue-white light; RBY, red-blue-yellow light; W, white light. Red text indicates up-regulated DEGs; green text indicates down-regulated DEGs.

|  | **Term** | **Pathway ID** | **DEG identifier** |
| --- | --- | --- | --- |
| **RB Vs RBG** | Amino sugar and nucleotide sugar metabolism | map00520 | BnaA03g20340D; BnaA06g02800D; BnaC01g41980D; BnaC03g24360D; BnaC04g10300D; BnaC06g03640D; BnaC06g03650D; BnaCnng22570D; MSTRG.26615 |
|  | Galactose metabolism | map00052 | BnaA01g37220D; BnaA06g02800D; BnaC06g03640D; BnaC06g03650D |
|  | Glycerophospholipid metabolism | map00564 | BnaA03g02290D; BnaA06g08550D; BnaC04g33210D; BnaC05g00450D; BnaC05g09920D |
|  | Phosphonate and phosphinate metabolism | map00440 | BnaA06g08550D; BnaC05g09920D |
|  | Fructose and mannose metabolism | map00051 | BnaA06g02800D; BnaC01g41980D; BnaC06g03640D; BnaC06g03650D |
|  | Starch and sucrose metabolism | map00500 | BnaA01g37220D; BnaA06g02800D; BnaC04g03740D; BnaC06g03640D; BnaC06g03650D; BnaA04g19070D; BnaCnng71250D |
|  | Plant hormone signal transduction | map04075 | BnaA09g50600D; BnaC03g71460D; BnaC05g14720D; BnaA01g04710D; BnaA03g51960D; BnaA08g05350D; BnaA08g30190D; BnaA09g56620D; BnaA10g16830D; BnaA10g16840D; BnaAnng36550D; BnaC01g06240D; BnaC03g43120D; BnaC08g09640D |
|  | Nitrogen metabolism | map00910 | BnaA07g36300D; BnaC02g06690D; BnaC03g03050D; BnaC07g13050D |
|  | Glycosphingolipid biosynthesis - lacto and neolacto series | map00601 | BnaA05g29310D; BnaCnng24210D |
|  | Glycosphingolipid biosynthesis - globo and isoglobo series | map00603 | BnaA05g29310D; BnaCnng24210D |
|  | Steroid biosynthesis | map00100 | BnaA09g41120D; BnaC01g03770D；BnaA02g37020D |
|  | Sesquiterpenoid and triterpenoid biosynthesis | map00909 | BnaC01g03770D；BnaA02g37020D |
|  | Glucosinolate biosynthesis | map00966 | BnaAnng06570D; BnaC04g29320D |
|  | Glycosaminoglycan degradation | map00531 | BnaA04g05180D; BnaA04g05190D; BnaC02g04940D |
|  | Ribosome | map03010 | BnaA04g05330D; BnaA05g33020D; BnaA05g34140D; BnaA05g34620D; BnaA08g06730D; BnaAnng34360D; BnaAnng37490D; BnaC01g38310D; BnaC01g44960D; BnaC02g01730D; BnaC02g02550D; BnaC02g03430D; BnaC02g05260D; BnaC02g05940D; BnaC08g34270D; BnaC08g49400D; BnaCnng24330D; BnaCnng58960D; MSTRG.28680; MSTRG.48821 |
|  | Tropane, piperidine and pyridine alkaloid biosynthesis | map00960 | BnaAnng01570D; BnaAnng01590D; BnaC04g14140D; BnaC04g14160D |
|  | Oxidative phosphorylation | map00190 | BnaA05g32350D; BnaA08g21880D; BnaC02g01030D; BnaC02g01040D; BnaC02g04740D; BnaC02g04750D; BnaC02g04760D; BnaA01g30740D; BnaA01g30750D; BnaA09g00900D; BnaA09g51600D |
| **RB Vs RBW** | ABC transporters | map02010 | BnaC01g17890D; BnaC01g38010D; BnaA05g26160D; BnaA10g01190D; BnaC05g01280D |
|  | Glycosphingolipid biosynthesis - ganglio series | map00604 | BnaA04g05180D; BnaA04g05190D； |
|  | Ribosome biogenesis in eukaryotes | map03008 | BnaA09g03590D; BnaC02g01270D; BnaC02g06220D; BnaC02g06980D; BnaC02g07930D; BnaC02g40470D; BnaC03g08830D； |
|  | Flavone and flavonol biosynthesis | map00944 | BnaA10g23330D |
|  | Circadian rhythm - plant | map04712 | BnaA10g19670D; BnaC02g01170D; BnaC02g03470D; BnaCnng20200D |
|  | Valine, leucine and isoleucine biosynthesis | map00290 | BnaA09g55140D; BnaA09g55150D; BnaCnng64850D |
|  | Lysine biosynthesis | map00300 | BnaA10g20730D; BnaA10g20750D |
| **RB Vs RBY** | Galactose metabolism | map00052 | BnaA04g05180D; BnaA04g05190D |
|  | Glycosaminoglycan degradation | map00531 | BnaA04g05180D; BnaA04g05190D |
|  | Oxidative phosphorylation | map00190 | BnaA08g21880D; BnaC02g13180D; BnaC05g48790D; BnaCnng18330D; MSTRG.17; MSTRG.41305 |
|  | Ascorbate and aldarate metabolism | map00053 | BnaC07g40160D; BnaC02g13320D; BnaC02g13330D; BnaC02g13690D |
|  | mRNA surveillance pathway | map03015 | BnaA07g10940D; BnaC04g27210D; BnaC04g27220D; BnaC04g27230D; BnaC05g47890D; BnaC07g10740D |
|  | Glycosphingolipid biosynthesis -ganglio series | map00604 | BnaA04g05180D; BnaA04g05190D |
|  | Phagosome | map04145 | BnaA08g21880D; BnaC02g13180D; BnaC03g04140D; BnaC05g48790D; MSTRG.41305 |
|  | Sulfur metabolism | map00920 | BnaA04g06360D; BnaA05g34080D; BnaA05g37220D; BnaC04g38860D |
|  | Other glycan degradation | map00511 | BnaA04g05180D; BnaA04g05190D |
|  | Biotin metabolism | map00780 | BnaC02g32230D; MSTRG.55717 |
|  | Sphingolipid metabolism | map00600 | BnaA04g05180D; BnaA04g05190D |
|  | Various types of N-glycan biosynthesis | map00513 | BnaA05g16320D; BnaC02g05850D |
|  | alpha-Linolenic acid metabolism | map00592 | BnaA06g06290D; BnaC03g56590D |
|  | Mismatch repair | map03430 | BnaAnng40820D; BnaA08g31450D; BnaAnng28510D; MSTRG.26231 |
| **RB Vs W** | Amino sugar and nucleotide sugar metabolism | map00520 | BnaA05g33470D; BnaC02g06080D; BnaC02g06160D; BnaA03g32270D; BnaC03g37570D; BnaC03g37610D; BnaC04g39360D; BnaC09g51720D; MSTRG.33463 |
|  | Sesquiterpenoid and triterpenoid biosynthesis | map00909 | BnaAnng36420D; BnaC07g30440D; BnaC07g30450D; BnaCnng41750D |
|  | Linoleic acid metabolism | map00591 | BnaA07g19600D; BnaC06g18870D |
|  | Mismatch repair | map03430 | BnaAnng40820D; BnaC01g44340D; BnaC02g18200D; BnaC07g19520D; BnaAnng25730D; BnaAnng28000D; MSTRG.26231; MSTRG.31249 |
|  | Nucleotide excision repair | map03420 | BnaAnng25730D; BnaAnng28000D; BnaAnng33860D; MSTRG.26231; MSTRG.31249; BnaAnng40820D; BnaC01g44340D; BnaC02g18200D; BnaC07g19520D |
|  | MAPK signaling pathway - plant | map04016 | BnaC04g53510D; BnaA03g32270D; BnaA06g14510D; BnaC03g37570D; BnaC03g37610D; MSTRG.33463 |
|  | Cutin, suberine and wax biosynthesis | map00073 | BnaC04g45430D; BnaC09g36490D |
|  | Homologous recombination | map03440 | BnaA05g33420D; BnaAnng40820D; BnaC01g44340D; BnaC02g18200D; BnaC07g19520D; BnaAnng25730D; BnaAnng28000D; MSTRG.26231; MSTRG.31249 |
|  | Oxidative phosphorylation | map00190 | BnaA05g32320D; BnaA05g32350D; BnaA08g21880D; BnaA10g22770D; BnaA10g22920D; BnaC02g04740D; BnaC02g04750D; BnaC02g04760D; BnaC07g41190D; BnaC09g01430D; BnaCnng16210D |
|  | Ascorbate and aldarate metabolism | map00053 | BnaA10g21300D |
|  | Phagosome | map04145 | BnaC03g04140D; BnaA08g21880D |
|  | alpha-Linolenic acid metabolism | map00592 | BnaA07g19600D; BnaC06g18870D |

**Table S5 The primer sequences of target and reference genes used in RT-qPCR analysis**

| **Transcript ID** | **Gene name** | **Sequence length (bp)** | **Primer sequence** | | **Amplicon size(bp)** |
| --- | --- | --- | --- | --- | --- |
|  |  |  | **Forward sequence (5’-3’)** | **Reverse sequence (5’-3’)** |  |
| MSTRG.41186 | *BnaC05g48840D* | 792 | AAGACGCACCTCATCAAC | CAGCCAGTGTCATTACCA | 112 |
| MSTRG.54219 | *BnaCnng42590D* | 831 | GGTTACCCACAGCTTCACGA | AGCAAGAGCAAACCCAACCT | 217 |
| MSTRG.32678 | *BnaC03g23750D* | 429 | TGTTCTCAGTGGGATTGG | TGTTGCCGTCACCTTTAT | 149 |
| MSTRG.42082 | *BnaC06g14680D* | 633 | GCGAAAGCGAAAGCCTATGG | TGTTGTGCTCAGCCTGGAAT | 110 |
| MSTRG.42081 | *BnaC06g14690D* | 1062 | GGTCGCTTCTGTAACTGG | TGCGGTCAATAAGGTAGG | 90 |
| MSTRG.29356 | *BnaC02g06430D* | 3312 | GAGTCGGTCCACATCCTG | GAACTTCCTCGCAAATAAAA | 149 |
| MSTRG.7677 | *BnaA03g56750D* | 429 | TGTTCTCATTGGGATTGG | TGTTGCCGTCACCTTTAT | 149 |
| MSTRG.2899 | *BnaA02g10970D* | 1659 | ACGAGATGCTGCTTACGA | CGAGACGGGAGTGTTCAG | 91 |
| MSTRG.42906 | *BnaC06g30100D* | 264 | GACTCATCATTGGCTACTGC | CATCTCCTCCACCACTCC | 96 |
| MSTRG.8840 | *BnaA04g17820D* | 1416 | GCTTTGCGGTGTTTCTAT | CAATGCGTTTGTCTCCTG | 148 |
| MSTRG.18428 | *BnaA08g28590D* | 1377 | AGGTCCTTCATGCTCATC | GACTAAGCCCATCGGTAT | 137 |
| MSTRG.12828 | *BnaA06g17310D* | 975 | GTTCTCCTCTATGCCTCC | CCACAGTTATTACCTCGTC | 284 |
| MSTRG.45987 | *BnaC07g43320D* | 855 | ACCAATCAAGGGCTCACC | CTCCCATTCATAAGTCACGATA | 130 |
| MSTRG.14137 | *BnaA06g37870D* | 975 | ATGAACGGCGGAGGAAAG | TAGCCACGGAGGAGCAAA | 89 |
| MSTRG.11569 | *BnaA05g34150D* | 1590 | CGCATCCAATCCTCGCCTCA | AGCCGTCCCGTTCGTCTCGT | 179 |
| MSTRG.12296 | *BnaA06g08550D* | 1152 | TCATCAATGGACCTACAGA | GGCACCCAACTAAACAAT | 124 |
|  | *actin-2* | 1131 | TAACCCAAAGGCTAACAGA | GAATCCAGCACAATACCG | 135 |
